# Neutralization of Hv1/HVCN1 With Antibody Enhances Microglia/Macrophages Myelin Clearance by Promoting Their Migration in the Brain

**DOI:** 10.3389/fncel.2021.768059

**Published:** 2021-10-22

**Authors:** Fan Wang, Xiao-Ru Ma, Yang Wu, Yong-Cheng Xu, Hui-Min Gu, Di-Xian Wang, Zhao-Jun Dong, Hui-Liang Li, Li-Bin Wang, Jing-Wei Zhao

**Affiliations:** ^1^Department of Pathology and Department of Human Anatomy, Histology and Embryology, Sir Run Run Shaw Hospital, System Medicine Research Center, NHC and CAMS Key Laboratory of Medical Neurobiology, Zhejiang University School of Medicine, Hangzhou, China; ^2^Division of Medicine, Wolfson Institute for Biomedical Research, University College London, London, United Kingdom; ^3^The General Hospital of Ningxia Medical University, Yinchuan, China

**Keywords:** voltage-gated proton channel, Hv1/HVCN1, voltage sensor domain only protein/VSOP, microglia/macrophages, migration, myelin phagocytosis, expression

## Abstract

Microglia dynamically monitor the microenvironment of the central nervous system (CNS) by constantly extending and retracting their processes in physiological conditions, and microglia/macrophages rapidly migrate into lesion sites in response to injuries or diseases in the CNS. Consequently, their migration ability is fundamentally important for their proper functioning. However, the mechanisms underlying their migration have not been fully understood. We wonder whether the voltage-gated proton channel HVCN1 in microglia/macrophages in the brain plays a role in their migration. We show in this study that in physiological conditions, microglia and bone marrow derived macrophage (BMDM) express HVCN1 with the highest level among glial cells, and upregulation of HVCN1 in microglia/macrophages is presented in multiple injuries and diseases of the CNS, reflecting the overactivation of HVCN1. In parallel, myelin debris accumulation occurs in both the focal lesion and the site where neurodegeneration takes place. Importantly, both genetic deletion of the HVCN1 gene in cells *in vitro* and neutralization of HVCN1 with antibody in the brain *in vivo* promotes migration of microglia/macrophages. Furthermore, neutralization of HVCN1 with antibody in the brain *in vivo* promotes myelin debris clearance by microglia/macrophages. This study uncovers a new role of HVCN1 in microglia/macrophages, coupling the proton channel HVCN1 to the migration of microglia/macrophages for the first time.

## Introduction

Microglia account for a proportion of total cells in the central nervous system (CNS), 5–10% in rodents and 16% in human (Lloyd and Miron, 2019). They play key roles in maintaining the homeostasis of the CNS by immune surveillance, phagocytic scavenging, and inflammation orchestration (Li and Barres, 2018). As resident immune cells in the CNS, in physiological condition, microglia constantly survey brain activity by extending and retracting their ramified processes to detect microenvironmental cues (Nimmerjahn et al., 2005), and microglia repopulate themselves effectively in a manner of self-renewal (Huang et al., 2018a, b; Xu et al., 2020). Microglial dysregulation and activation are implicated in almost all injuries and diseases of the CNS. In response to CNS injury, both CNS resident microglia and blood monocyte-derived macrophages are rapidly recruited to the lesion site, where both of them are activated as microglia/macrophages (Li and Barres, 2018). In other words, microglia/macrophages have dual origin in injured or diseased CNS (Bennett and Bennett, 2020; Prinz et al., 2021) and it is hard to distinguish between them. Therefore, we use the phrase microglia/macrophages to describe them in contexts of CNS injury or disease. Therefore, the motility or migration ability of microglia is fundamentally important for their functioning, especially in pathological conditions.

Accumulating evidence has shown that microglia/macrophages can play detrimental or beneficial roles in context-dependent manners in injured or diseased CNS (Hickman et al., 2018). Upon demyelinated injury, microglia/macrophages can promote remyelination in the CNS by accelerating the clearance of myelin debris. Within demyelination lesion of both patients with multiple sclerosis (MS) and experimental animal models of demyelination, myelin debris accumulates and inhibits the recruitment and differentiation of oligodendrocyte progenitor cell (OPC) (Kotter et al., 2006; Neumann et al., 2009). With progressive aging, the capacity of microglia/macrophages to phagocytose myelin declines, and this decline is positively correlated with a decrease in remyelination efficiency. Remarkably, promoting myelin debris clearance ability of microglia/macrophages in the aged CNS by rejuvenating microglia/macrophages through parabiosis (Ruckh et al., 2012) or by regulating their activation states (Miron et al., 2013) eventually enhances remyelination efficiency. Therefore, promoting the ability of microglia/macrophages in myelin debris clearance represents an effective strategy to enhance remyelination (Lloyd and Miron, 2019).

The essential functions of microglia/macrophages, migration, phagocytosis, and inflammation largely depend on their membrane potential and ion channels (Izquierdo et al., 2019). The hydrogen voltage-gated channel 1, Hv1/HVCN1 (also known as voltage sensor domain only protein, VSOP), has been shown to be expressed not only in microglia of the CNS (Wu et al., 2012; DeCoursey, 2018; He et al., 2021) but also in peripheral immune cells (DeCoursey, 2018). Using transgenic *Hvcn1* knockout (*Hvcn1^–/–^*) mice in which the *Hvcn1* gene is deleted, many studies have shown that loss of the proton channel HVCN1 exhibits neuroprotective effects in CNS injury models, such as stroke (Wu et al., 2012), traumatic brain injury (Ritzel et al., 2021), and spinal cord injury (Li et al., 2021). Interestingly, genetic deletion of *Hvcn1* decreases the demyelination lesion volume in both focal and systemic demyelination models (Liu et al., 2015; Chen et al., 2020). So far, the mechanisms underlying the protective effects of systematically genetic deletion of *Hvcn1* have mainly focused on reducing the reactive oxygen species (ROS) in microglia (DeCoursey et al., 2003; Kawai et al., 2017; Luo et al., 2021). Up to date, it remains elusive whether HVCN1 is also expressed on other types of glial cells in the CNS, and it is unknown whether the expression pattern of HVCN1 is conserved across different species. More importantly, considering that migration and phagocytosis are fundamentally important for microglia/macrophages to function efficiently, we ask whether the voltage-gated proton channel HVCN1 in microglia/macrophages in the brain plays a role in their migration and phagocytosis of myelin.

In this study, we identified a specific anti-HVCN1 antibody and showed that among glial cells, HVCN1 was predominantly expressed in microglia in the brain of mouse, marmoset, and human. HVCN1 was also expressed in OPC and mature oligodendrocyte in the brain albeit at a much lower level. In multiple injuries and diseases of the CNS, we found upregulation of HVCN1 in microglia/macrophages, reflecting its overactivation. Functionally, genetic deletion of *HVCN1* in cultured cells revealed that HVCN1 inhibits cellular migration *in vitro*. Furthermore, neutralization of HVCN1 with antibody in the brain promoted microglia migration and enhanced myelin debris clearance *in vivo*. Our results uncovered a new role of HVCN1 in microglia, coupling the proton channel HVCN1 to microglia migration for the first time.

## Materials and Methods

### Human Brain Tissue

Postmortem human brain tissues were obtained from National Health and Disease Human Brain Tissue Resource Center^1^ and were approved by the Medical Ethics Review Committee of Zhejiang University School of Medicine. The detailed information of human samples is shown in Supplementary Table 1.

### Marmoset Brain Tissue

The brain tissues of two healthy marmosets were obtained from Dr. Li-Xia Gao’s laboratory: one is a male aged 96 months (M) and the other is a female aged 62 months. All experiments with marmosets followed the guidelines approved by the Experimental Animal Welfare Ethics Review Committee of Zhejiang University (Zhejiang University Ethical Approval project number: ZJU20190079).

### Mice

All animal experiments were performed according to the guidelines of the Institutional Animal Care and Use Committee of Zhejiang University and approved by the Ethics Committee of the Zhejiang University School of Medicine. Wild type (WT) C57BL/6 mice and SD (Sprague Dawley) rats were obtained from Shanghai SLAC (slaccas) Laboratory Animal Co., Ltd. (Shanghai, China). SJL (SJL/JOrlIcoCrl) male mice (Charles River, Beijing, China) were crossed with female C57BL/6 mice to get B6SJL mice. *SOD1*^*G*93*A*^ mice (Jackson Laboratory, ME, United States, 002726) were bred with B6SJL female mice. R6/2 mice (027421) were obtained from Jackson Laboratory (ME, United States). *Hvcn1*^*fl/fl*^ mice (T019403) were obtained from GemPharmatech, Co., Ltd. (Nanjing, China). G3 *Terc^–/–^* mice (Telomerase RNA component knockout mice, third generation) were obtained from Dr. Zhen-Yu Ju laboratory and genotyped using PCR reactions with three primers to amplify the WT and knockout alleles (1, 5′-TTCTGACCACCACCAACTTCAAT-3′, 2, 5′-GGGGCTGCTAAAGCGCAT-3′, 3, 5′-CTAAGCCG GCACTCCTTACAAG-3′). The sizes of PCR products of WT and *Terc^–/–^* alleles are 220 and 180 bp, respectively. All mice were kept in the Laboratory Animal Center of Zhejiang University under a 12 h light/dark cycle, with food and water *ad libitum*.

### Cell Line Culture and Transfection

COS-7, 293T, and Hela cells were cultured in Dulbecco’s modified essential media (DMEM, HyClone, UT, United States, SH30022.01) supplemented with 10% fetal bovine serum (FBS, Biological industries, Kibbutz Beit-Haemek, Israel, 04-001-1ACS).

For primary microglia culture, the cerebral cortex of the P0 SD rat was dissociated, minced, and digested with 0.25% trypsin. The mixture was transferred to DMEM complete media (DMEM supplemented with 10% FBS and 1% penicillin/streptomycin) and pipetted rigorously several times to dissolve any small pieces. After standing still for 5 min, the suspension was filtered through 70 μm nylon sieves and centrifuged at 500 *g* for 5 min. The precipitate was resuspended with DMEM complete media and plated into poly-D-lysine (PDL, Sigma, P6407) coated 75 cm^2^ flasks. After 8-day-maintenance, microglia were isolated by shaking on a rotary shaker at 200 rpm for 2 h at 37°C. Floating cells were harvested and plated on PDL-coated culture dishes or coverslips.

For primary bone marrow derived macrophage (BMDM) culture, bone marrows of tibiae and femurs from 10–12 weeks male C57BL/6 mice were flushed with PBS (phosphate buffered saline). Cells were filtered through 70 μm nylon sieves. Erythrocytes were lysed with red blood cell lysis buffer. Cells were cultured in DMEM media supplemented with 10% FBS, 1% penicillin/streptomycin, and 10 ng/ml M-CSF (macrophage colony stimulating factor, Pepro Tech, NJ, United States, 315-02) for 5–7 days.

Lipofectamine 2000 (Thermo, MA, United States, 11668-019) was used for transient transfection of plasmid DNA and siRNA (small interfering RNA) into 293T, Hela, and COS-7 cells. Lipofectamine RNAi (Thermo, 13778-075) was used for transient transfection of siRNA into primary cultured microglia and BMDM cells.

### Plasmids and siRNAs

Plenti-CMV-mCherry, Plenti-CMV-HVCN1-HA-mCherry, Plenti-CMV-HVCN1-HA-p2A-mCherry, and Plenti-CMV-HVCN1-HA-GFP were purchased from WZ Biosciences Inc (Jinan, China). The TMEM192-GFP plasmid was obtained from the laboratory of Dr. Wei Liu. All siRNA oligomers used in this study were synthesized by GenePharma (Shanghai, China). The sequences are as follows: siControl, 5′-UUCUCCGAACGUGUCACGUTT-3′ and 5′-ACGUGACACGUUCGGAGAATT-3′; siHVCN1-1, 5′-C CGUCACUCGCAGAACCAATT-3′ and 5′-UUGGUUCUGC GAGUGACGGTT-3′; siHVCN1-2, 5′-CCCACAGGUUUCA GGUCAUTT-3′ and 5′-AUGACCUGAAACCUGUGGGTT-3′; siRNA-3, 5′-GGAGGGUGGCCCGGAUCAUTT-3′ and 5′-AUGAUCCGGGCCACCCUCCTT-3′. Cy3 conjugated siNC and siHVCN1-2 were used for transfection in primary cultured microglia and BMDM cells.

### Construction of *HVCN1* Knockout Cells

Two guide RNAs (gRNAs), 5′-GAACTTGCTCATCCTCTCAG-3′ and 5′-ACCCACACCAGTCTCAGGCG-3′, were cloned into lenti-CRISPRv2-Puro and transfected into 293T cells and Hela cells. After being selected with 2 μg/ml puromycin for 3 days, cells were diluted into single clones in 96-well plates. Genomic PCR and anti-HVCN1 immunoblotting were used to screen *HVCN1^–/–^* clones. The PCR primer used are: F1, 5′-AATAAAAGCACGAGAAACCACC-3′; R1, 5′-CTGAACAGTTTCCTCAACATGC-3′; R2, 5′-CTT GTAGTTGATGTTCCAGGCA-3′. The genomic PCR products of positive cell clones were ligated to pCE2-TA vector by TA/Blunt-Zero Cloning Kit (Vazyme, Nanjing, China, C601-01) and transformed into DH5α *E. coli*. For each cell clone, at least 10 bacteria clones were evaluated by PCR and DNA sequencing.

### Myelin Isolation and Labeling

Myelin was isolated as previously reported (Norton and Poduslo, 1973). In brief, rats were euthanized and their brains were homogenized in ice-cold 0.32 M sucrose with a Dounce tissue grinder set (Sigma, D9188). The homogenates were layered over 0.85 M sucrose and centrifuged at 75,000 *g* for 30 min. The interface layer of the two sucrose solutions was collected and washed with water three times, centrifuged one time at 75,000 *g* for 15 min and two times at 12,000 g for 10 min. The precipitate was resuspended in PBS and Myelin protein content was determined by BCA (bicinchoninic acid) assay.

Myelin (8 mg protein/ml) was labeled with 5 μM carboxyfluorescein succinimidyl ester (CFSE, BD biosciences, NJ, United States, 565082) or 10 μM pHrodo iFL Green STP ester (pHrodo, Thermo, P36013) for 30 min at 37°C. Excessive dye was removed by washing with PBS three times. Labeled myelin was resuspended with PBS and stored in aliquots at –80°C.

### Tissue Preparation

Mice were anesthetized with intraperitoneal (i.p.) injection of pentobarbital sodium and intracardially perfused with PBS followed by 4% paraformaldehyde (PFA). Targeted tissue was isolated, postfixed, and cryoprotected in 30% sucrose until it sank to the bottom. The tissue was taken out and embedded in OCT (optimal cutting temperature compound, SAKURA, CA, United States, 4583). After OCT solidification at –20°C, tissue was cut with a cryostat (Leica, Wetzlar, Germany, CM1950) into sections at a thickness of 12 μm and stored at –80°C.

### Virus/LPC/Myelin Injection

Mice were anesthetized with an i.p. injection of pentobarbital sodium, and they were then placed in a stereotaxic apparatus. The skull above the target area was exposed and a hole was drilled above the injection site. Viruses, LPC (lysophosphatidylcholine), or myelin were injected with a 10 μl Hamilton syringe (NV, United States) for 5 min. After each injection, the needle stayed still for 5 min and was then slowly withdrawn.

For virus injection, AAV2/9-CamKIIα-GFP or AAV2/9-CamKIIα-cre-p2A-GFP were obtained from WZ Biosciences Inc. (Jinan, China). 0.2 μl virus was injected into the primary motor cortex (coordinates: 0.5 mm anterior, 1.0 mm lateral, 1.25 mm ventral relative to Bregma). Mice were sacrificed as described 4 weeks postinjection.

For LPC injection, 1% LPC (Sigma, L4129) was sonicated and then injected into the corpus callosum (coordinates: 0.5 mm anterior, 0.0 mm lateral, 2.3 mm ventral relative to Bregma) for 1 μl per mouse. Mice were sacrificed as described 5 days postinjection.

Myelin injections were performed as previously reported (Pluvinage et al., 2019). Briefly, CFSE-labeled myelin (25 mg/ml) was boiled for 10 min followed by cooling on ice immediately to denature it. The degraded myelin was mixed with 1 mg/ml rabbit Immunoglobulin G (IgG) isotype as control antibody (Sino Biological, Beijing, China, CR1) or Rabbit anti-HVCN1 (Origene, TA328862). One μl IgG or antibody-myelin mixture was injected into the primary sensory (S1) cortex (coordinates: 0.0 mm anterior, ±2 mm lateral, 1.5 mm ventral) on one side of hemispheres of mice, respectively. Mice were sacrificed as described 48 h postinjection.

### Lipopolysaccharide Injury

Mice received an i.p. injection of 5 mg/kg ultrapure *E. coli* 0111: B4 lipopolysaccharide (LPS) (Invivogen, CA, United States, trl-pelps) diluted in 0.9% sterile saline or saline alone. Mice were sacrificed as described 12 h postinjection.

### Immunohistochemistry

Slides were air-dried, washed three times in PBS for 5 min each, and blocked with 5% donkey serum in PBS containing 0.3% Triton X-100 (Sigma, MO, United States, T8787) for 2 h at room temperature. These two primary antibodies were applied for 48–72 h at designed dilution: rat anti-CD68 (Abcam, Cambridge, United Kindom, ab53444, 1:200) and rabbit anti-degraded MBP (Millipore, MA, United States, AB5864, 1:4000). For the following antibodies, sodium citrate buffer microwave antigen retrieval was done before the block: Goat anti-Iba1 (Abcam, ab5076, 1:500), biotin-IB4 (Sigma, L2140, 1:500), mouse anti-iNOS (BD sciences, 610329, 1:100), goat anti-Arginase 1 (Santa Cruz, TX, United States, sc-18355, 1:100), chicken anti-GFAP (Millipore, AB5541, 1:1000), goat anti-SOX10 (R & D systems, MN, United States, AF2864, 1:200), goat anti-GFP (Abcam, ab5450, 1:1000), and rabbit anti-HVCN1 (Origene, MD, United States, TA328862, 1:100). After rinsing for three times in PBS for 5 min each, slides were incubated with the following fluorescently conjugated secondary antibodies (1:500) for 2 h at 4°C: Alexa Fluor 488-conjugated donkey anti-mouse (Jackson ImmunoResearch, PA, United States, 715-545-150), Alexa Fluor 488-conjugated donkey anti-goat (Jackson ImmunoResearch, 705-545-003), Alexa Fluor 488-conjugated donkey anti-chicken (Jackson ImmunoResearch, 703-545-155), Alexa Fluor 488-conjugated streptavidin (Jackson ImmunoResearch, 016-540-084), Cy3-conjugated donkey anti-rat (Jackson ImmunoResearch, 712-165-153), Cy3-conjugated donkey anti-rabbit (Jackson ImmunoResearch, 711-165-152), Alexa Fluor 647-conjugated donkey anti-rabbit (Jackson ImmunoResearch, 711-605-152), and Alexa Fluor 647-conjugated streptavidin (Jackson ImmunoResearch, 016-600-084). Following incubation, washing was carried out three times in PBS and counterstaining with DAPI, (4’,6-diamidino-2-phenylindole) slides were rinsed three times in PBS and mounted under coverslips with FluorSave reagent (Millipore, 345789). Images were taken by Olympus BX53 or FV1000 confocal microscope.

### Immunocytochemistry

Cells plated on coverslips were washed with PBS and then fixed with 4% PFA for 10 min at room temperature. After washing with PBS, coverslips were blocked with 5% donkey serum in PBS containing 0.3% Triton X-100 for 1 h at room temperature. Primary antibodies were incubated for 1∼3 h at room temperature at the following dilution, rat anti-CD11b (AbD serotec, Kidlington, United Kingdom, MCA711, 1:100) and rabbit anti-HVCN1 (Origene, TA328862, 1:100). Coverslips were washed with PBS, and incubated with the following fluorescently conjugated secondary antibody (1:500) for 0.5∼1 h at room temperature: Alexa Fluor 647 conjugated donkey anti-rat (Jackson ImmunoResearch, 712-605-153, 1:500), Cy3-conjugated donkey anti-rat (Jackson ImmunoResearch, 712-165-153), and Cy3-conjugated donkey anti-rabbit (Jackson ImmunoResearch, 711-165-152). Coverslips underwent a wash in PBS and were stained with DAPI for 10 min at room temperature. After the final wash in PBS, coverslips were mounted onto microscopy slides with FluorSave mounting medium (Millipore, 345789). Images were taken by Olympus BX53 or FV1000 confocal microscope.

### *In vitro* Assay for Myelin Phagocytosis

Cells were seeded in coverslips or culture dishes for at least 24 h for further experiments. CFSE-myelin and pHrodo-myelin were diluted in DMEM and incubated at 37°C for 1 h. The media of cells were replaced with myelin-containing DMEM and incubated at 37°C, cells were washed with ice-cold PBS five times. For immunofluorescence, cells were fixed with 4% PFA, rinsed with PBS, and stored in PBS. For flow cytometry, cells were scraped on ice, resuspended in ice-cold PBS. After filtering through 70 μm nylon sieves, cells were measured on an FC500 cytometer (Beckman Coulter, CA, United States) or a CytoFLEX LX flow cytometer (Beckman Coulter). Data were evaluated with flowjo software. The percentage of cells phagocytizing myelin was calculated. The phagocytic index (PI) was defined as the accumulated fluorescence of phagocytotic cells divided by total cell number. The PI was normalized to the corresponding control group.

### Transwell Assay

Cells were washed with DMEM media and resuspended in DMEM media containing 0.2% FBS at a density of 2 × 10^5^ cells/ml. The upper chamber of the transwell plates (Corning, NY, United States, 3422) was plated with 200 μl cell suspension, and the lower chamber was added with 600 μl DMEM media supplemented with 10% FBS. After 24 h incubation, the upper chambers were taken out, rinsed in PBS, and fixed in 4% PFA for 10 min at room temperature. The cells on the top side of the chamber were wiped. The chambers were stained with 0.1% crystal violet and rinsed with water. Images were taken under an Olympus BX53 microscope.

### Western Blot

To wash away the blood, animals were intracardially perfused with PBS under deep anesthesia with pentobarbital sodium. Tissues were isolated, minced, and sonicated in lysis III buffer (50 mM Tris-Cl pH 7.4, 50 mM NaCl, 5 mM EDTA (ethylene diamine tetraacetic acid), 60 mM CHAPS (3-[(3-cholamidopropyl)-dimethyl-ammonio]-1-propane sulfonate), 0.5% sodium deoxycholate, 1% SDS (sodium dodecyl sulfate), 1% NP-40, 1% Triton X-100) supplemented with 1 mM DTT (dithiothreitol), protease inhibitor cocktail (Roche Life Science, 4693132001), phosphatase inhibitor cocktail (Biomake, TX, United States, B15001), and 2 μM MG132 (Millipore, 474790). Cultured cells were harvested, washed with PBS, and lysed in lysis III buffer on ice for 30 min. Lysates were centrifuged at 12,000 g for 10 min at 4°C. Supernatants were collected and the protein concentrations were evaluated with a BCA assay. Protein samples were loaded for SDS-PAGE and transferred to 0.2 μm polyvinylidene difluoride membranes (Millipore, ISEQ00010). Membranes were blocked with 5% non-fat milk in TBST (tris buffered saline with 0.5% Tween-20) for 2 h at room temperature. Primary antibodies were incubated in 3% non-fat milk in TBST overnight at 4°C. Primary antibodies used were as follows: Rabbit anti-HVCN1 (Origene, TA328862, 1:1000), horseradish peroxidase (HRP) conjugated mouse anti-HA (Millipore, H6533, 1:10, 000), mouse anti-β-tubulin (HUABIO, Hangzhou, China, M1305-2, 1:2000), mouse anti-β-actin (Sigma, A5441, 1:10,000). After washed with TBST buffer three times, membranes were incubated with HRP conjugated secondary antibodies (1:10, 000) for 2 h at room temperature. Secondary antibodies used were as follows: HRP conjugated Donkey anti-rabbit (Jackson ImmunoResearch, 711-035-152), HRP conjugated Donkey anti-mouse (Jackson ImmunoResearch, 715-035-151). Immunoreactivity was visualized by FDbio-Dura ECL (electrochemiluminescence, Fdbio science, Hangzhou, China, FD8020) and detected by ChemiDoc Touch Imaging System (Bio-Rad).

The postmortem human brain tissues that were stored at –80°C, were from National Health and Disease Human Brain Tissue Resource Center. Total protein extraction was performed by the identical protocol as the animal protein described.

### Reverse Transcription-Quantitative PCR

Total RNA was isolated using TRIzol reagent (Invitrogen, CA, United States, 15596-018). Reverse transcription (RT) was performed using HiScript II Q RT SuperMix (Vazyme, Nanjing, China, R223-01). Quantitative PCR (qPCR) was carried out using 2 × T5 Fast qPCR Mix (Tsingke, Hangzhou, China, TSE202) with CFX96 Touch (Bio-Rad, CA, United States). ΔΔCt values normalized to *Gapdh* were used to evaluate relative gene expression between samples. The primers used for *Hvcn1* are 5′-CCAAGAGGATGAGCAGGTTCTTGAA-3′ (forward) and 5′-CAGGACCACCAGGCAGATGATG-3′ (reverse) and those for *Gapdh* are 5′-AGGTCGGTGTGAACGGATTTG-3′ (forward) and 5′-TGTAGACCATGTAGTTGAGGTCA-3′ (reverse).

### RNA Sequencing

The RNA sequencing (RNA-seq) service was provided by Beijing Genomics Institute (BGI, Shenzhen, China). Briefly, RNA was isolated from WT or *HVCN1* knockout 293T cells using TRIzol reagent (Invitrogen, 15596-018). The mRNA was purified utilizing poly-T oligo attached magnetic beads and then fragmented into small pieces. The cleaved RNA fragments were reverse transcribed into first-strand cDNA using random primers. After amplification and quality control of the cDNA libraries were performed, paired-end sequencing (2 × 150 bp) was performed *via* the BGISEQ-500 platform (BGI). The sequence data were filtered with SOAPnuke (v1.5.2), mapped to the human reference genome GRCh38.p12 using HISAT2 (v2.0.4). Bowtie2 (v2.2.5) was applied to align the clean reads to the gene set. The transcription level of genes was calculated by RSEM (RNA-Seq by Expectation Maximization, v1.2.12) and quantified as transcripts per kilobase million (TPM).

### Statistics

Data were presented as mean ± SEM. Statistical tests were one-way ANOVA with Tukey’s or Dunnett’s multiple comparison posttests, two-way ANOVA with Sidak’s multiple comparison posttest, paired or unpaired two-tailed *t*-test using Graphpad Prism software, and *p* < 0.05 was taken as statistically significant. Statistical methods, results, and sample size related to each figure are presented in Supplementary Table 2.

## Results

### Expression of HVCN1 in Microglia *in vitro* and in the Normal Adult Cerebral Cortex of Mouse, Marmoset, and Human

**FIGURE 1:**
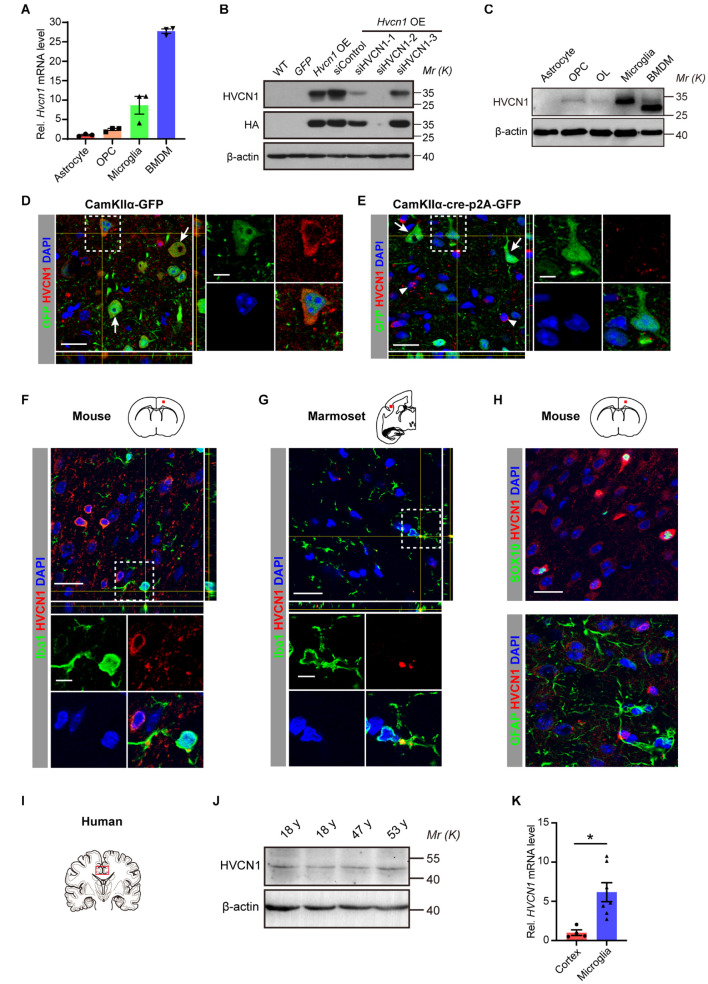
Expression of HVCN1 in microglia *in vitro* and in the normal adult cerebral cortex of mouse, marmoset, and human. **(A)** *Hvcn1* mRNA is highly expressed in primary cultured mouse bone marrow derived macrophages (BMDM) and rat microglia, while exhibits much lower level in oligodendrocyte progenitor cells (OPCs) and astrocyte of the rat brain (*n* = 3). **(B)** Specificity validation of the HVCN1 antibody *in vitro* by Western blot and *Hvcn1* is overexpressed (OE) by transfecting with pLenti-CMV-HVCN1-HA-p2A-GFP plasmid whereas knocked down with siHVCN1 in COS-7 cells. **(C)** Representative images of Western blot of primary cultured cells: HVCN1 protein is expressed highly in rat microglia and mouse BMDM while exhibits low levels in rat OPCs, oligodendrocyte (OL), and astrocyte. **(D,E)** Specificity validation of HVCN1 antibody *in vivo* by immunofluorescence: targeted deleting *Hvcn1* in CamKIIα^+^ neurons of *Hvcn1*^*fl/fl*^ mice by focal injection of AAV9-CamKIIα-Cre-p2A-GFP virus into the cerebral cortex. In GFP^+^ (green, CamKIIα^+^) neurons in the cortex, immunofluorescence of HVCN1 (red) exhibits abundant signal in control **(D)** while eliminates labeling after *Hvcn1* is targeted deleted **(E)**. As examples, arrows indicate GFP^+^ (CamKIIα^+^) neurons, while arrowheads indicate CamKIIα negative cells. **(F,G)** Representative immunofluorescence images: HVCN1 (red) is expressed in Iba1^+^ microglia (green) in the cerebral cortex of mouse **(F)** and marmoset **(G)**. **(H)** Immunofluorescence images: HVCN1 (red) is expressed in a few SOX10^+^ oligodendrocytes (green, upper) but not in GFAP^+^ astrocyte (green, lower) in the cerebral cortex of the mouse. **(I)** Schematic drawing shows the site of human brain samples. **(J)** Representative Western blot images of HVCN1 in the postmortem human brain. **(K)** *HVCN1* mRNA level in the cortex of the postmortem human brain (*n* = 4) and the microglia isolated from the human brain (*n* = 7) of patients with epilepsy (Gosselin et al., 2017). Scale bars **(D–H)** = 20 μm; the side length of the dashed-line square = 5 μm. Data are presented as mean ± SEM. Statistical tests: unpaired two-tailed *t*-test **(K)**; *p < 0.05.

It has been shown that HVCN1 is expressed in microglia in the mouse brain (Wu et al., 2012). However, it remains elusive whether other glial cells also express HVCN1. To compare the relative abundance of *Hvcn1* at mRNA level among glial cells, primary glial cells and OPC were cultured from the cerebral cortex of newborn rat (P0) and primary BMDM was cultured from the bone marrow of the femur and tibia of the adult mouse. Our RT-qPCR data showed that in terms of the abundance of *Hvcn1* mRNA, among the four types of tested cells, BMDM exhibits the most abundant level, and microglia have the second-highest level and also rank the highest among the two types of glial cells and OPC in the brain. OPC has a much lower level while astrocyte has very little and is the lowest among the four cell types (Figure 1A). To compare the protein levels of HVCN1, a specific anti-HVCN1 antibody was identified. We selected the COS-7 cell as a cell model since its WT form does not express HVCN1 protein (Zeng and Xu, 2012). *Hvcn1* was overexpressed (OE) by transfecting pLenti-CMV-HVCN1-HA-p2A-GFP, and based on the *Hvcn1* OE, *Hvcn1* was further knocked down with small interference RNA, siHVCN1, in COS-7 cells. In combination with the HA tag, Western blot using the HVCN1 antibody verified that the anti-HVCN1 antibody (referred to as “the HVCN1 Ab1” throughout this study) is specific to HVCN1 (Figure 1B). Armed with this specific antibody directed to HVCN1, the HVCN1 Ab1, we tested the HVCN1 protein level in the primary cultured cells by Western blot. As shown in Figure 1C, we confirmed that, in consistent with our RT-PCR data, at the protein level, HVCN1 is expressed most highly in microglia and BMDM while exhibits a low level in OPC, oligodendrocyte (OL), and none in astrocyte.

To further characterize the specificity of the HVCN1 Ab1 *in vivo*, we performed immunofluorescence staining using a conditional *Hvcn1* knockout mouse by focal injection of virus in the cortex. We injected the control virus, CamKIIα-GFP adeno-associated virus (AAV2/9), into the cortex of adult *Hvcn1*^*fl/fl*^ mice and found that the immunofluorescence signal of HVCN1 was detected in CamKIIα positive neurons (Figure 1D). In contrast, after targeted knocking out *Hvcn1* by injecting the AAV2/9-Cre virus, AAV2/9-CamKIIα-Cre-p2A-GFP, into the cortex of *Hvcn1*^*fl/fl*^ mice, the immunofluorescence signal of HVCN1 was abolished in CamKIIα positive neurons infected with AAV2/9, while still remained in some CamKIIα negative cells (Figure 1E). These results indicate that the HVCN1 Ab1 specifically detects HVCN1 in the brain *in vivo*. To investigate what types of cells in the mouse brain express HVCN1, we performed immunofluorescence labeling using the HVCN1 Ab1, and observed that HVCN1 was expressed in a small proportion of Iba1^+^ microglia in the primary motor cortex (M1) of the mouse (Figure 1F) and in the secondary somatosensory cortex (S2) of marmoset (Figure 1G). The HVCN1 was also expressed in some SOX10^+^ oligodendrocyte lineage cells but not in GFAP^+^ astrocytes (Figure 1H). By using Western blot, we showed that HVCN1 protein was expressed in the cingulate cerebral cortex of postmortem adult human participants (Figures 1I,J). To clarify whether human microglia also express HVCN1, we extracted and analyzed the *HVCN1* data from the RNA-seq data library, deposited by Gosselin et al. (2017), in which they performed RNA-seq of both the cortex and the acutely sorted microglia from patients with epilepsy. This analysis confirmed that *HVCN1* mRNA is highly expressed in the microglia of the human brain (Figure 1K). Collectively, HVCN1 is highly expressed in microglia *in vivo* across species from mouse, marmoset, to human.

Aging is a major risk factor for many neurodegenerative diseases (Hou et al., 2019). We wonder whether the expression level of HVCN1 in the brain changes with progressive aging. To detect the protein level of HVCN1 in the whole brain (from the olfactory bulb to the oblongata) of mice, by using Western blot with the HVCN1 Ab1, we showed that with progressive aging, the HVCN1 protein level in the brain did not change either in the naturally aging mice (Supplementary Figures 1A,B) or in the premature aging G3 *Terc^–/–^* mice (Telomerase RNA component knockout mice, third generation) (Supplementary Figures 1C,D). Furthermore, in the postmortem cingulate cerebral cortex of human subjects, whose diagnosis was excluded from CNS-relevant tumors or diseases, the protein level of HVCN1 in the human cingulate cerebral cortex also did not change with progressive aging (Supplementary Figures 1E,F). These results indicate that the protein level of HVCN1 in the brain does not change with progressive aging in both mice and humans.

### Upregulation of HVCN1 in Microglia/Macrophage in Multiple Injuries and Diseases of the Central Nervous System

**FIGURE 2:**
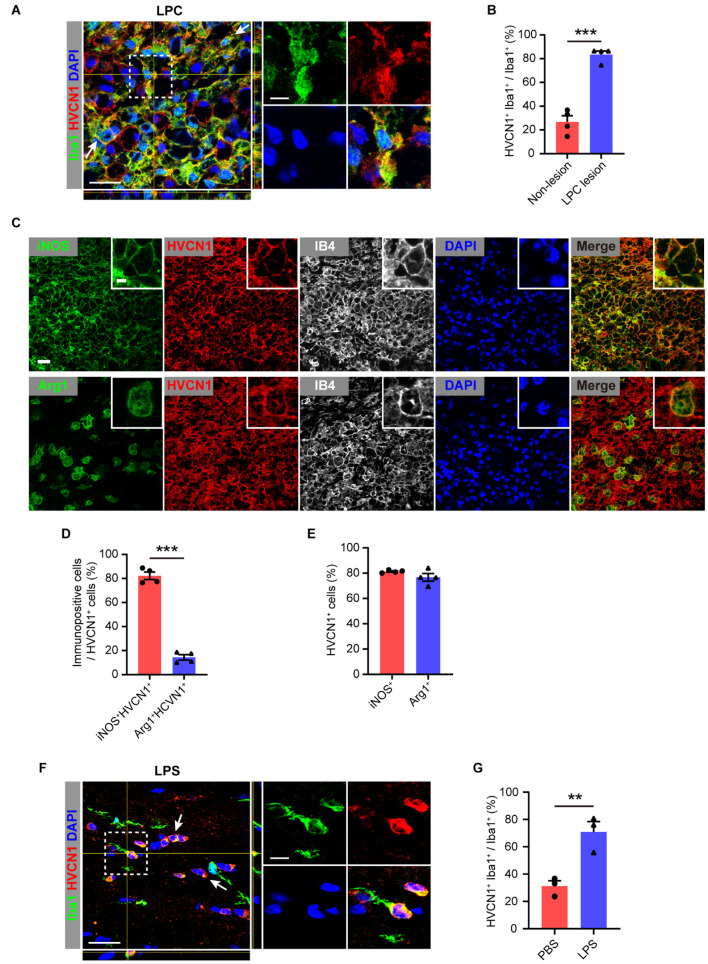
Upregulation of HVCN1 in microglia/macrophage in multiple injuries. Representative immunofluorescence images **(A)** and quantification of the proportion **(B)**: HVCN1 (red) in Iba1^+^ (green) microglia/macrophages in focal demyelination lesion in corpus callosum of the mouse brain at 5 days after focal injection of lysolecithin (LPC). (*n* = 4). Two HVCN1^+^Iba1^+^ microglia are indicated as examples. **(C)** Immunofluorescence images show that HVCN1 (red) is expressed in both iNOS^+^ (green, up) type and Arg1^+^ (green, down) type polarized IB4^+^ microglia/macrophages (gray). **(D)** The proportion of iNOS^+^ and Arg1^+^ type microglia/macrophages in HVCN1^+^ cells (*n* = 4/group). **(E)** The proportion of HVCN1^+^ cells in iNOS^+^ and Arg1^+^ type microglia/macrophages (*n* = 4/group). **(F,G)** Representative immunofluorescence images **(E)** and quantification of the proportion **(F)**: HVCN1 (red) in Iba1^+^ (green) microglia/macrophage in corpus callosum of mouse brain at 12 h after intraperitoneal (i.p.) injection of lipopolysaccharide (LPS). (*n* = 3/group). Two HVCN1^+^Iba1^+^ microglia are indicated as examples. Scale bars **(A,C,E)** = 20 μm; the side length of the dashed-line square = 5 μm. Data are presented as mean ± SEM. Statistical tests: unpaired two-tailed *t*-test **(B,D,F)**; **p < 0.01, ***p < 0.001.

Since microglia plays an important role in various injuries and diseases of CNS (Greenhalgh et al., 2020), we wonder whether the expression of HVCN1 in microglia is changed in injuries and diseases of CNS. To test whether the expression of HVCN1 is changed in a focal lesion in the CNS, we induced focal demyelination by injection of lysolecithin (LPC) into the corpus callosum of adult WT mice. We found at 5 days postlesion (dpl), the proportion of Iba1^+^ microglia/macrophages, which expressed HVCN1 in all microglia/macrophages within the focal demyelination lesion in the corpus callosum, upregulated to four times of that for the non-lesion WT mice (Figures 2A,B). Similar results were obtained when IB4, another marker of microglia/macrophage, was used (Supplementary Figure 2). Within the focal demyelinated lesion, HVCN1 was expressed in a few oligodendrocytes but not in astrocytes (Supplementary Figure 2). Previously we showed that Arg1^+^ microglia promote myelin repair in demyelination lesions (Miron et al., 2013). We wonder whether the increased expression of HVCN1 in microglia/macrophages is different between iNOS^+^ and Arg1^+^ types. We showed that among HVCN1^+^ microglia/macrophages, the proportion of iNOS^+^ microglia/macrophages were about 80%, whereas the proportion Arg1^+^ microglia/macrophages were about 15% (Figures 2C,D), indicating that within the focal demyelination lesion, the upregulated HVCN1 was mainly in iNOS^+^ microglia/macrophages. Of note, the proportion of HVCN1^+^ cells among either iNOS^+^ or Arg1^+^ microglia/macrophages were equivalent, roughly 80% for both (Figure 2E), suggesting no preference between iNOS^+^ and Arg1^+^ microglia/macrophages.

To further test whether the expression of HVCN1 in the brain is affected by a systemic injury, which also involves the brain, we induced systemic inflammation in adult mice by an i.p. injection of LPS and 12 h later when brain inflammation has been established (Zhao et al., 2009). Our immunofluorescent results showed that in comparison with the PBS-treated mice, the proportion of HVCN1 positive microglia among all microglia in the corpus callosum significantly increased in the LPS-treated mice (Figures 2F,G). Together, these results indicate that HVCN1 is upregulated in microglia/macrophages of the brain not only within focal demyelination lesion but also in the brain of systemic inflammation.

**FIGURE 3:**
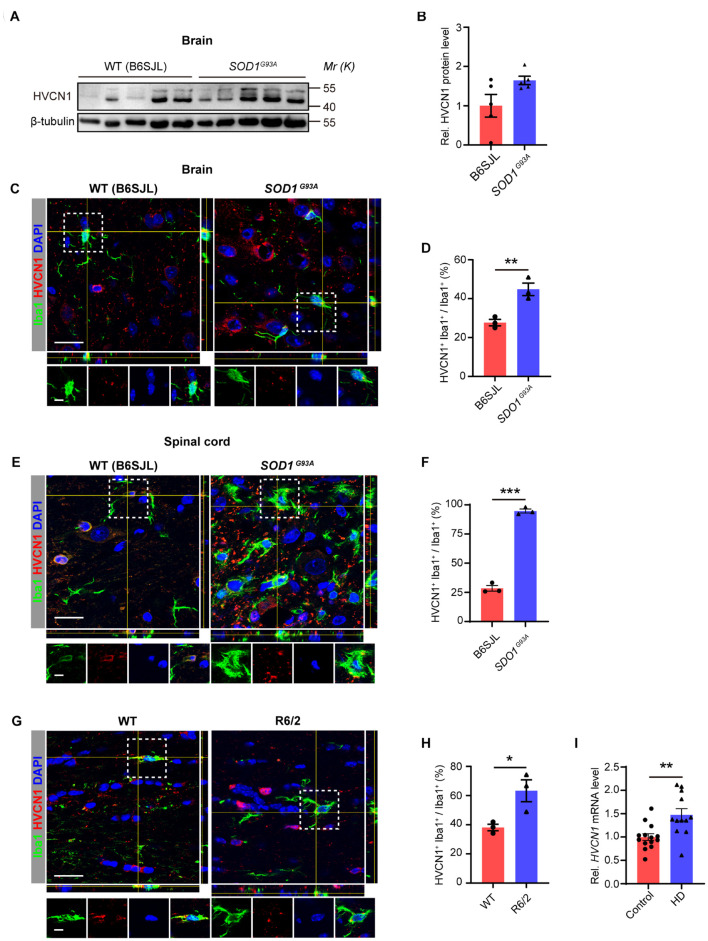
Upregulation of HVCN1 in microglia/macrophage in neurodegenerative diseases of the central nervous system. **(A,B)** Representative Western blot images **(A)** and quantification of densities **(B)** of HVCN1 in the whole brain of *SOD1*^*G*93*A*^ mice and their littermates B6SJL mice (*n* = 5). **(C–F)** Representative immunofluorescence images **(C,E)** and quantification of the proportion **(D,F)**: HVCN1 (red) is expressed in Iba1^+^ microglia/macrophages (green) in the brain **(C,D)** and the spinal cord **(E,F)** of *SOD1*^*G*93*A*^ mice and their littermates B6SJL mice (*n* = 3). **(G,H)** Representative immunofluorescence images **(G)** and quantification of the proportion **(H):** HVCN1 (red) is expressed in Iba1^+^ microglia/macrophages (green) in the brain of wild type (WT) and R6/2 mouse (*n* = 3). **(I)** *HVCN1* mRNA level in the peripheral blood of patients with Huntington’s Disease (*n* = 14) in comparison with the healthy control subjects (*n* = 12) (Borovecki et al., 2005). Scale bars **(C,E,G)** = 20 μm; the side length of the dashed-line square = 5 μm. Data are presented as mean ± SEM. Statistical tests: unpaired two-tailed *t*-test **(B,D,F,H,I)**; *p < 0.05, **p < 0.01, ***p < 0.001.

To explore whether the protein level of HVCN1 is affected in neurodegenerative diseases, we first detected the HVCN1 protein level in *SOD1*^*G*93*A*^ mice, a mostly used mouse model of amyotrophic lateral sclerosis (ALS). We found that in comparison with the control mice, there was a trend of increase in HVCN1 protein level in the whole brain (from the olfactory bulb to the oblongata) of *SOD1*^*G*93*A*^ mice (Figure 3A), despite there is no significant difference (Figure 3B). Immunofluorescence data revealed that a significant increase in the proportion of microglia/macrophages that expressed HVCN1 was detected both in the cerebral cortex of the brain (Figures 3C,D) and the anterior horn of the spinal cord of SOD1^*G*93*A*^ mice (Figures 3E,F). Remarkably, over three times increase was seen in the anterior horn of the spinal cord where the key pathology for ALS resides (Figure 3F). To test whether HVCN1 is upregulated in other neurodegenerative diseases, using R6/2 mice, the mouse model of Huntington’s disease (HD), we found that in the cerebral cortex, the proportion of microglia/macrophages that expressed HVCN1 in all microglia/macrophages doubled (Figures 3G,H). Importantly, we analyzed the RNA-seq data deposited by Borovecki et al. (2005) and found that in peripheral blood of patients with HD, the mRNA level of HVCN1 was significantly increased (Figure 3I). Taken together, these results indicate that HVCN1 is upregulated in microglia/macrophage in multiple injuries and neurodegenerative diseases of the CNS.

### *HVCN1* Genetic Deletion *in vitro* Promotes Cell Migration

**FIGURE 4:**
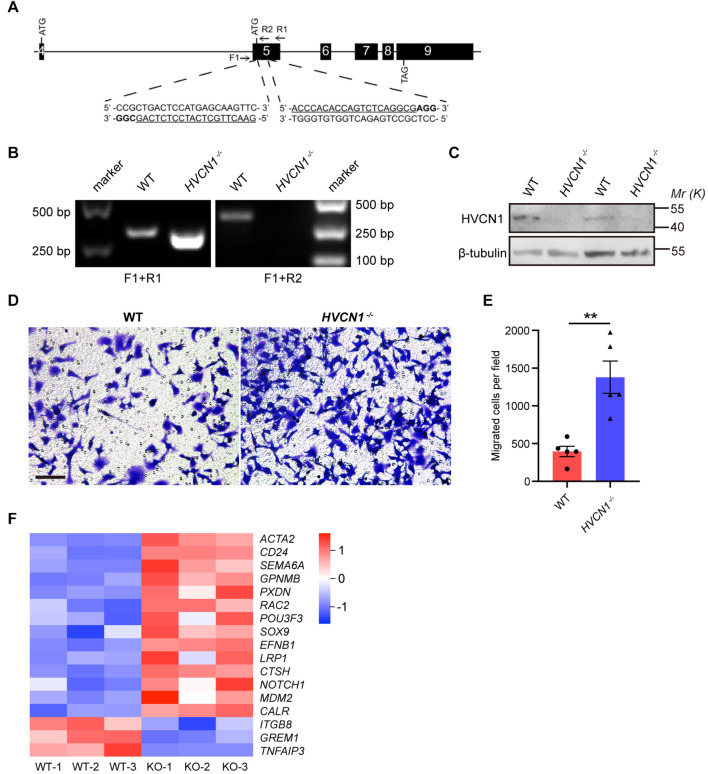
*HVCN1* genetic deletion *in vitro* promotes cell migration. **(A)** *HVCN1* knockout strategy by CRISPR/Cas9 in Hela cells. Black boxes, exons; line, introns; Arrows, primers for PCR detection; Underlined, sgRNA targeted sequences; Bold, protospacer adjacent motif (PAM). **(B)** Genomic PCR products of WT and *HVCN1^–/–^* Hela cells. The position of primers is shown in **(A)**. **(C)** Western blot images of HVCN1 in WT and *HVCN1^–/–^* Hela cells. **(D)** Representative images of transwell assay. Scale bars = 100 μm. **(E)** Quantification of transwell assay (*n* = 5). **(F)** A heatmap of migration related gene expression changes between WT and *HVCN1*^–/–^ 293T cells by RNA-seq. Data are presented as mean ± SEM. Statistical tests: unpaired two-tailed *t*-test **(E)**; ** *p* < 0.01.

Having shown that HVCN1 is upregulated in microglia in multiple brain injuries and diseases, we next investigate the role of HVCN1 in microglia. It has been established that the major functional aspects of microglia in the CNS include migration (Kreutzberg, 1996), phagocytosis (Fu et al., 2014), and inflammation (Cunningham, 2013). So far, as the mechanisms underlying the effects of HVCN1 have been focused on ROS (Wu et al., 2012), we wonder whether HVCN1 affects cell migration. To explore this, we knocked out *HVCN1* in Hela cells using the CRISPR-Cas9 technique (Figure 4A). The deletion of *HVCN1* in Hela cells was verified by PCR at the genomic level (Figure 4B) and by Western blot at protein level (Figure 4C). Transwell migration assay was used to compare the *in vitro* migration ability between the *HVCN1^–/–^* and the WT control Hela cells. Our results showed that genetic deletion of *HVCN1* increased the number of Hela cells migrating from serum-free medium to serum-containing medium to about three times that of the WT control Hela cells (Figures 4D,E), indicating that genetic deletion of *HVCN1* promotes cell migration *in vitro*. We also performed bulk RNA-seq in *HVCN1^–/–^* and WT 293T cells. Our data showed that a series of genes that promote cell migration were upregulated: *ACTA2*, *CD24*, *SEMA6A*, *GPNMB*, *PXDN*, *RAC2*, *POU3F3*, *SOX9*, *EFNB1*, *LRP1*, *CTSH*, *NOTCH 1*, *MDM2*, and *CALR*; while three genes that inhibit cell migration were downregulated: *ITGB8*, *GREM1*, and *TNFAIP3* (Figure 4F). Taken together, our results revealed that genetic deletion of *HVCN1* promotes cell migration *in vitro*.

### Neutralization of HVCN1 With Antibody Promotes Cell Migration *in vivo*

**FIGURE 5:**
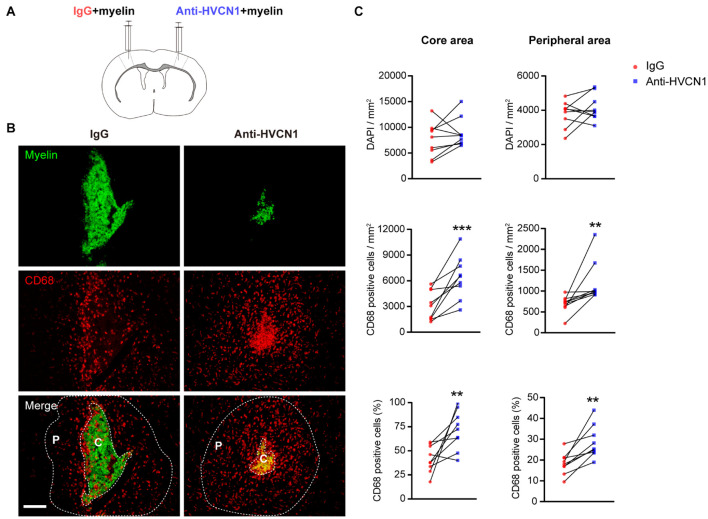
Neutralization of HVCN1 with antibody promotes cell migration *in vivo*. **(A)** Schematic *in vivo* experiment design: CFSE-labeled myelin was co-injected with IgG on one side or with an HVCN1 antibody on the other side of the S1 cerebral cortex of the mouse. **(B)** Representative images show that neutralization with HVCN1 antibody increases CD68^+^ cell (red) density in both the core area (C) and the peripheral area (P) of the myelin (green) injection site. **(C)** Quantification of CD68^+^ microglia/macrophages density in the core area (left column) and the peripheral area (right column) of the myelin injection site (*n* = 9). Scale bar **(B)** = 100 μm. Data are presented as mean ± SEM. Statistical tests: paired two-tailed *t*-test **(C)**; **p < 0.01, ***p < 0.001.

To further test whether HVCN1 regulates migration of microglia *in vivo*, we purified myelin from the adult rat brain (Norton and Poduslo, 1973) and labeled the myelin with CFSE. CFSE-labeled myelin was co-injected with IgG on one side while with the HVCN1 Ab1 on the other side of the S1 cerebral cortex of an adult mouse (Figure 5A). We found that at 48 h postinjection, in comparison with the IgG injection side, neutralization with HVCN1 antibody increases CD68^+^ microglia/macrophages (red) in both the core area (C) and the peripheral area (P) of the myelin injection site both in density and in proportion (Figures 5B,C), indicating that neutralization of HVCN1 with the HVCN1 Ab1 *in vivo* promotes migration of microglia. Taking our *in vitro* and *in vivo* data together, we show for the first time that both genetic deletion of *HVCN1 in vitro* and neutralization of HVCN1 with antibody *in vivo* promotes cell migration.

### Both Genetic Deletion and Overexpression of *HVCN1* Do Not Affect Phagocytosis of Myelin in 293T Cells

**FIGURE 6:**
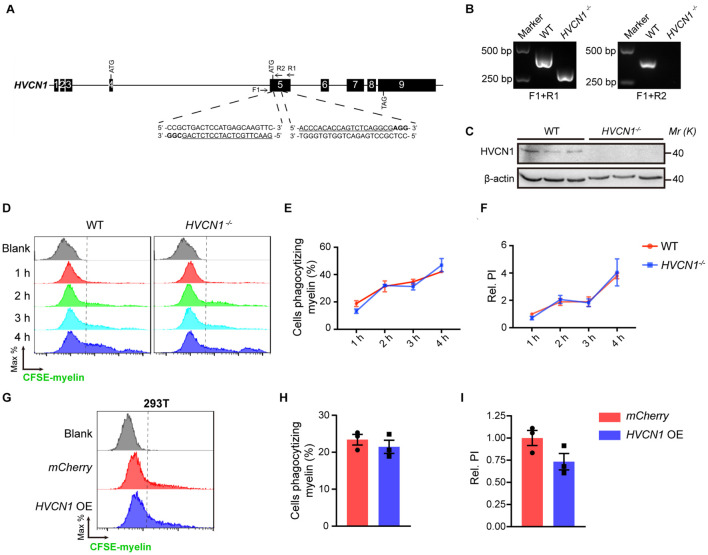
Both genetic deletion and overexpression of *HVCN1* do not affect phagocytosis of myelin in 293T cells. **(A)** *HVCN1* gene knockout strategy by CRISPR/Cas9 in 293T cells. Black boxes, exons; line, introns; Arrows, primers for PCR detection; Underlined, sgRNA targeted sequences; Bold, PAM. **(B)** Genomic PCR products of WT and *HVCN1^–/–^* 293T cells. The position of primers is shown in **(A)**. **(C)** Western blot images of HVCN1 in WT and *HVCN1^–/–^* 293T cells. **(D–F)** Flow cytometry chart **(D)** and the quantification graphs **(E,F)**: *HVCN1* knockout does not affect both the proportion of cells phagocytizing myelin **(E)** and the relative phagocytic index **(F)** in CFSE-labeled-myelin phagocytosis assay in 293T cells (*n* = 3). **(G–I)** Flow cytometry chart **(G)** and the quantification graphs **(H,I)** show that overexpression of *HVCN1* does not affect both the proportion of cells phagocytizing myelin **(H)** and the relative phagocytic index **(I)** in CFSE-labeled-myelin phagocytosis assay in 293T cells (*n* = 3). Data are presented as mean ± SEM. Statistical tests: two-way ANOVA with Sidak’s multiple comparison posttest **(E,F)** or unpaired two-tailed *t*-test **(H,I)**.

So far, it is unknown whether HVCN1 affects phagocytosis (He et al., 2021). To explore this, we knocked out *HVCN1* in 293T cells using the CRISPR-Cas9 technique (Figure 6A). The deletion of *HVCN1* was verified by PCR at the genomic level (Figure 6B) and Western blot at protein level (Figure 6C). To compare the phagocytic ability between the *HVCN1^–/–^* and the WT 293T cells, we added CFSE-labeled myelin to the culture medium and detected the fluorescence intensity within the cells by using flow cytometry. Our data showed that in comparison with the WT 293T cells, *HVCN1* knockout does not affect the proportion of cells phagocytizing myelin (Figures 6D,E). To accurately calculate the phagocytic capacity of cells, we defined the PI as the accumulated fluorescence of phagocytic cells divided by the total number of cells. We found that the relative phagocytic index (PI) of the *HVCN1^–/–^* 293T cells did not change compared with the WT 293T cells (Figure 6F), and the phenomenon was prolonged up to 4 h (Figures 6D–F). Furthermore, overexpression of *HVCN1* in 293T cells did not affect either the proportion of cells phagocytizing myelin (Figures 6G,H) or the relative PI (Figures 6G,I). Our data indicate that both genetic deletion and overexpression of *HVCN1* do not affect phagocytosis of myelin in 293T cells.

### *HVCN1* Deletion Impairs Lysosome Acidification During Myelin Phagocytosis *in vitro*

**FIGURE 7:**
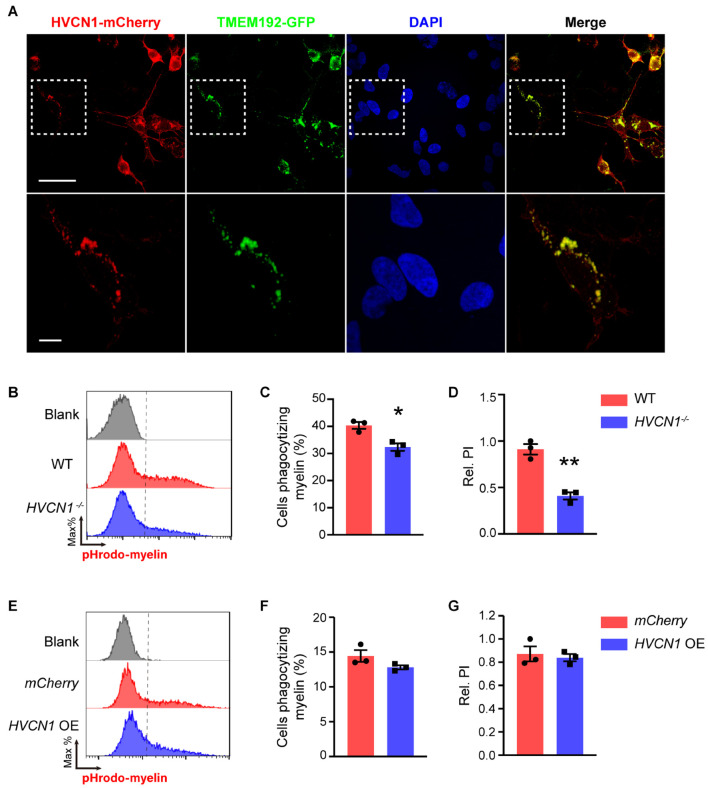
*HVCN1* deletion impairs lysosome acidification during myelin phagocytosis *in vitro*. **(A)** Representative images show that HVCN1 (red) is colocalized with lysosome marker TMEM192 (green) in 293T cells overexpressed Plenti-HVCN1-mCherry and TMEM192-GFP plasmid. Scale bar = 50 μm (upper row) or 10 μm (lower row). Flow cytometry chart **(B)** and the quantification graphs **(C,D)** show that *HVCN1* knockout decreases both the proportion of cells phagocytizing myelin **(C)** and the relative phagocytic index **(D)** in pHrodo-myelin phagocytosis assay in 293T cells (*n* = 3). Flow cytometry chart **(E)** and the quantification graphs **(F,G)** show that overexpression of *HVCN1* does not affect the proportion of phagocytic cells **(F)** and the relative phagocytic index **(G)** in pHrodo-myelin phagocytosis assay in 293T cell (*n* = 3). Data are presented as mean ± SEM. Statistical tests: unpaired two-tailed *t*-test **(C,D,F,G)**; *p < 0.05, **p < 0.01.

Since HVCN1 is expressed both on the plasma membrane and within cells, we wonder whether HVCN1 is expressed in the lysosome, and is involved in maintaining the lower pH in the lysosome. To test this possibility, using the genetic labeling technique, we visualized lysosomes with TMEM192-GFP, an established method to label the lysosome (Schroder et al., 2010), and HVCN1 with HVCN1-mCherry. Interestingly, we found *HVCN1* overexpressed in 293T cells was colocalized with the lysosome marker TMEM192 (Figure 7A), indicating that HVCN1 is expressed in the lysosome, an organelle which is closely involved in phagocytosis, especially the digestion process of cells (Flannagan et al., 2012). To test whether HVCN1 affects myelin in the lysosome, we labeled myelin with pHrodo, which shows a weak fluorescent signal at neutral pH while increasing its fluorescence intensity in a lower pH, therefore pHrodo exhibits strong signals in lysosome where pH is much lower than any other compartments of the cell. We found that *HVCN1* knockout decreased both the proportion of cells phagocytizing myelin (Figures 7B,C) and the relative PI (Figures 7B,D) in pHrodo-myelin phagocytosis assay in 293T cell, indicating that deletion of *HVCN1* impaired lysosome acidification in myelin containing cells, likely reflecting reduced digestion of myelin. However, overexpression *of HVCN1* did not affect either the proportion of cells phagocytizing myelin (Figures 7E,F) or the relative PI (Figures 7E,G) in pHrodo-myelin phagocytosis assay in 293T cell. These results indicate that HVCN1 is necessary but not sufficient to maintain acidic condition in lysosome during phagocytosis, suggesting the possibility that HVCN1 is involved in the digestion of myelin.

### Knockdown of *HVCN1* Does Not Affect Myelin Phagocytosis and Lysosome Acidification *in vitro*

**FIGURE 8:**
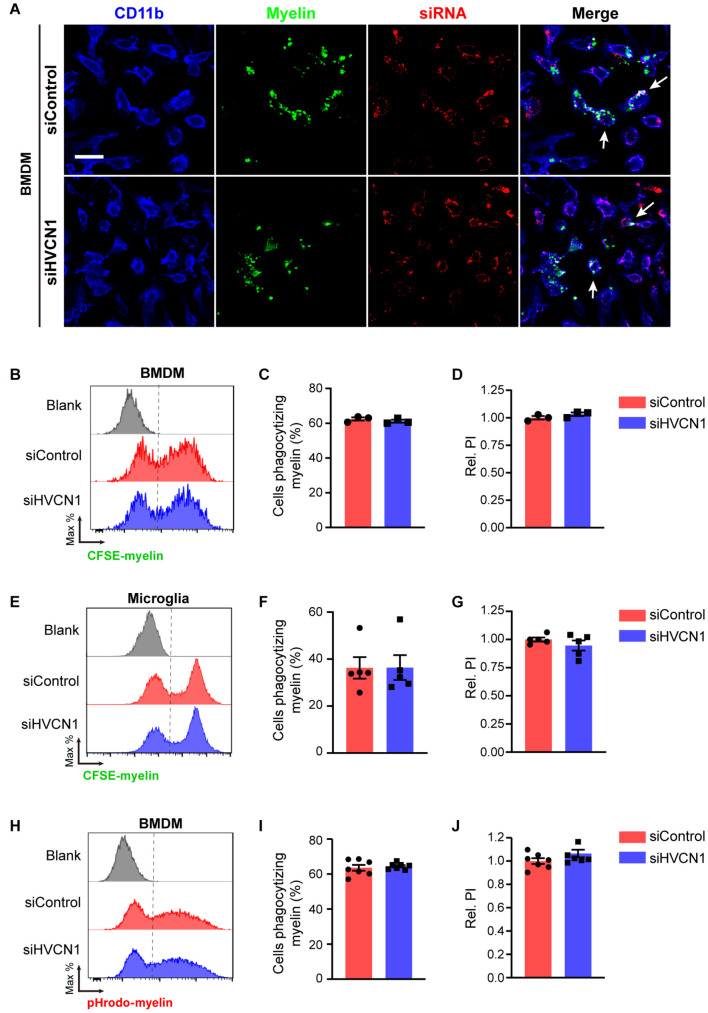
Knockdown of *HVCN1* does not affect myelin phagocytosis and lysosome acidification *in vitro*. **(A)** Representative immunofluorescence images show that *Hvcn1* knockdown by Cy3 labeled siRNA (red) does not affect myelin phagocytosis (green) in CD11b^+^ BMDM (blue). Scale bar = 100 μm. Arrows indicate two example BMDM cells that phagocytized myelin. Flow cytometry chart **(B)** and the quantification graphs **(C,D)** show that *Hvcn1* knockdown does not affect both the proportion of cells phagocytizing myelin **(C)** and the relative phagocytic index **(D)** in CFSE-labeled myelin phagocytosis assay in BMDM cells (*n* = 3). Flow cytometry chart **(E)** and the quantification graphs **(F,G)** show that *Hvcn1* knockout does not affect both the proportion of cells phagocytizing myelin **(F)** and the relative phagocytic index **(G)** in CFSE-labeled myelin phagocytosis assay in primary cultured microglia cells (*n* = 5). Flow cytometry chart **(H)** and the quantification graphs **(I,J)** show that *Hvcn1* knockdown does not affect the proportion of cells phagocytizing myelin **(I)** and the relative phagocytic index **(J)** in pHrodo-myelin phagocytosis assay in BMDM cells (*n* = 7). Data are presented as mean ± SEM. Statistical tests: unpaired two-tailed *t*-test **(C,D,F,G,I,J)**.

The phagocytic ability of non-professional phagocytes such as 293T cells is much lower than that of professional phagocytes such as macrophage and microglia (Arandjelovic and Ravichandran, 2015). We wonder whether HVCN1 affects the phagocytosis capacity of microglia/macrophage. Using primary cultured BMDM as a cell model, we first optimized the myelin doses (Supplementary Figures 3A–C) and myelin incubation time (Supplementary Figures 3D–F) for WT BMDM to phagocytize myelin. Our results showed that the optimal myelin dose is 20 μg/mL, while the suitable phagocytic time is 45 min. The knockdown efficiency of siHVCN1 was confirmed by Western blot (Figure 1B) and siHVCN1-2 exhibited almost knockout effect so siHVCN1-2 was used in the following knockdown experiments. Under the optimized conditions, we showed that knockdown of *HVCN1* did not affect either the proportion of cells phagocytizing myelin (Figures 8A–C) or the relative PI (Figures 8A,B,D) in CFSE-labeled myelin phagocytosis assay in BMDM cells transfected with Cy3 conjugated siRNA. Similarly, in primary cultured microglia, *HVCN1* knockdown did not affect either the proportion of cells phagocytizing myelin (Figures 8E,F) or the relative PI (Figures 8E,G) during phagocytosis of total myelin. These results are consistent with the phagocytosis results of total myelin in *HVCN1* knockout 293T cells.

To further test whether knockdown of *HVCN1* affects the acidification of lysosomes during phagocytosis of myelin, we did the phagocytosis assay utilizing pHrodo-labeled myelin in BMDM cells. We found that *HVCN1* knockdown did not affect either the proportion of cells phagocytizing myelin (Figures 8H,I) or the relative PI (Figures 8H,J) in pHrodo-labeled myelin in the lysosome of BMDM cells. These results indicate that knockdown of *HVCN1* does not affect either the phagocytosis of total myelin or the lysosome acidification during myelin phagocytosis. Taking the data of *HVCN1* knockout and overexpression into account, we propose that HVCN1 is necessary but not sufficient to maintain the lysosomal low pH during myelin phagocytosis.

### Myelin Debris Accumulates in Both Lesioned and Diseased Sites in the Central Nervous System

**FIGURE 9:**
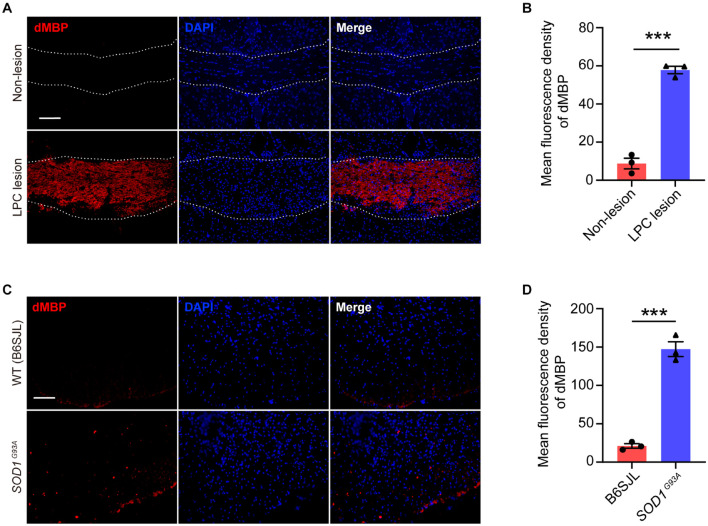
Myelin debris accumulates in both lesioned and diseased sites in the CNS. **(A,B)** Representative immunofluorescence images **(A)** and quantification of mean fluorescence intensity **(B)** of degraded myelin basic protein (dMBP, red) in the corpus callosum of the mouse brain of non-lesion (upper row) and LPC-induced demyelination lesion (lower row) (*n* = 3). **(C,D)** Representative immunofluorescence images **(C)** and quantification of mean fluorescence intensity **(D)** of dMBP (red) in the spinal cord of *SOD1*^*G*93*A*^ mice (lower row) and their littermates B6SJL mice (upper row) (*n* = 3). Scale bars = 100 μm **(A,C)**. Data are presented as mean ± SEM. Statistical tests: unpaired two-tailed *t*-test **(B,D)**; ***p < 0.001.

Myelin debris accumulation has been shown as a major pathology in demyelinated diseases, such as MS (Reich et al., 2018). In this study, we showed that myelin debris was seen within LPC-induced focal demyelinated lesions (Figures 9A,B). Importantly, we also observed a remarkable increase of degraded myelin in the ventral horn of the spinal cord of *SOD1*^*G*93*A*^ mice (Figures 9C,D), coinciding with the sites where spinal motor neuron degeneration occurs, suggesting that myelin debris accumulation is an obstacle to be overcome not only for demyelinated lesion but also for neurodegenerative diseases. Therefore, enhancing the efficiency of myelin debris clearance could be beneficial to ameliorate injuries or diseases of the CNS.

### Neutralizing HVCN1 With Antibody Enhances Myelin Debris Clearance in the Brain

**FIGURE 10:**
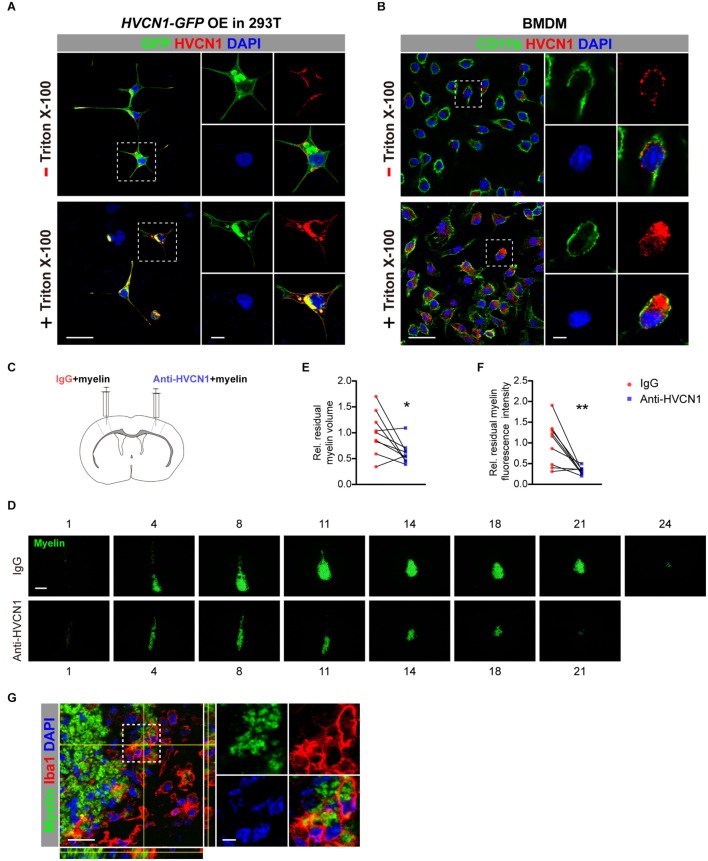
Neutralizing HVCN1 with antibody enhances myelin debris clearance in the brain. **(A,B)** The binding patterns of the HVCN1 antibody to cells without (upper row) or with Triton X-100 permeabilization (lower row). Confocal images of GFP (green), HVCN1 (red) in 293T cells overexpressed with Plenti-HVCN1-GFP **(A)**. Confocal images of CD11b (green), HVCN1 (red) in BMDM cells **(B)**. **(C)** Schematic *in vivo* experiment design: CFSE labeled myelin was co-injected with IgG on one side or with an HVCN1 antibody on the other side of the S1 cerebral cortex of the mouse. **(D–F)** Representative images of the residual myelin (green) taken in every 36 or 48 μm thickness of brain section **(D)** and quantification of its volume **(E)** and its fluorescence intensity **(F)** (*n* = 9). **(G)** Confocal images of the Iba1^+^ microglia (red), HVCN1 (gray), and phagocytized myelin (green). Scale bars = 100 μm **(D)** or 20 μm **(A,B,G)**, and the side length of the dashed-line square = 5 μm **(A,B,G)**. Data are presented as mean ± SEM. Statistical tests: paired two-tailed *t*-test **(E,F)**; *p < 0.05, **p < 0.01.

Having shown that HVCN1 is expressed both on the plasma membrane and in the cytoplasm, and upregulation of HVCN1 is seen in multiple injuries and diseases of CNS, suggesting that HVCN1 might be functionally overactivated. We wonder whether manipulating HVCN1, such as neutralizing HVCN1 with antibody, affects the ability of microglia on myelin debris clearance. To answer this question, we first tested whether the HVCN1 Ab1 binds to the extracellular side of the plasma membrane without cell membrane permeated by detergent. Using 293T cells overexpressed with HVCN1-GFP plasmid, we observed that without Triton X-100 permeabilization, the signal of the HVCN1 Ab1 was only detected at the extracellular side of the plasma membrane in 293T cells. In contrast, with Triton X-100 permeabilization, the signal of the HVCN1 Ab1 was seen both on the plasma membrane and in the cytoplasm (Figure 10A). Similar results were obtained in BMDM cells (Figure 10B). Collectively, these results showed that the HVCN1 Ab1 can bind to the extracellular side of the plasma membrane, suggesting that the HVCN1 Ab1 can be used as a neutralizing antibody.

Armed with this antibody, we ask whether neutralizing HVCN1 with the HVCN1 Ab1 affects myelin debris clearance in the brain. CFSE-labeled myelin purified from adult rat brain was co-injected with IgG in one side, while with the HVCN1 Ab1 in the other side of the S1 cerebral cortex of mouse (Figure 10C). We measured the area of residual myelin every 36∼48 μm and reconstructed the volume in a stereological way (Figure 10D). Our data showed that at 48 h postinjection, both the volume (Figure 10E) and the fluorescence intensity (Figure 10F) of residual myelin decreased in the side where the HVCN1 antibody was co-injected, indicating that myelin debris is cleared more effectively in the side of the HVCN1 Ab1. Indeed, the microglia/macrophage in the HVCN1 Ab1 co-injected side phagocytized myelin debris *in vivo* (Figure 10G). Quantitatively, microglia/macrophages occupied about 90% of the HVCN1^+^ cells (Supplementary Figure 4), suggesting that microglia/macrophage is the predominant cell type which is responsible for myelin debris clearance. Together, our results showed that neutralizing HVCN1 with antibody enhances myelin debris clearance in the brain *in vivo*.

## Discussion

Microglia dynamically monitor the microenvironment of the CNS by constantly extending and retracting their processes (Nimmerjahn et al., 2005), therefore, they are highly motile cells (Okochi et al., 2020). Consequently, their motility is fundamentally important for their proper functioning. The previous study has shown that HVCN1 is expressed in microglia in the mouse brain (Wu et al., 2012). To compare the relative abundance of HVCN1 in various types of glial cells at both mRNA and protein levels, we show here that microglia and BMDM exhibit the highest level of HVCN1 among all types of glial cells in the mouse brain. We further reveal that expression of HVCN1 in microglia conserves across species from mouse, marmoset, to human. Moreover, we report that upregulation of HVCN1 in microglia/macrophages is induced in multiple injuries and diseases of the CNS, suggesting that overactivation of HVCN1 in microglia/macrophages represents one of the common features on the reaction of microglia/macrophages to injuries or diseases in the CNS. Using *Hvcn1*^–/–^ mouse, previous studies have shown that genetic deletion of *Hvcn1* exhibits neuronal protection in various injuries in the CNS (Liu et al., 2015; Li et al., 2019b, 2020b; Chen et al., 2020; Murugan et al., 2020; Ritzel et al., 2021). Up to date, the mechanisms that underlie the effects of HVCN1 in the CNS have mainly focused on ROS in microglia: HVCN1 facilitates ROS production in physiological conditions, whereas it generates excessive ROS in pathological conditions (DeCoursey et al., 2003; Kawai et al., 2017; Li et al., 2020a; Murugan et al., 2020; Luo et al., 2021). To explore new roles of HVCN1, using transwell migration assay, we found that genetic deletion of *Hvcn1* promotes cell migration *in vitro*. To take a snapshot of transcriptional landscapes on the genetic deletion of *Hvcn1*, our bulk RNA-seq data show that genetic deletion of *Hvcn1* upregulated 14 and downregulated 3 cell migration-related genes. Among the upregulated genes, of note, LRP1 (low-density lipoprotein receptor-related protein 1) has been shown to promote OPC migration (Garcia-Fernandez et al., 2021), and SEMA6A (semaphorin 6A) is required for the migration of retinal progenitor cells (Belle et al., 2016) and cerebellar granule cells (Kerjan et al., 2005). Actin alpha 2 (ACTA2) (Xu et al., 2019), CD24 (Li et al., 2019a), glycoprotein nmb (GPnmb) (Taya and Hammes, 2018), and RAC2 (Rac family small GTPase 2) (Lu et al., 2020) have been reported to facilitate cell migration. The three downregulated genes, ITGB8, GREM1, and TNFAIP3 are also involved in cell migration (Wei et al., 2020). All these results consistently support that loss of HVCN1 promotes cell migration, providing clues to the underlying mechanisms. Our work couples the voltage-gated proton channel HVCN1 to microglia migration for the first time.

Until now, two potent inhibitors of HVCN1 have been identified [including 2-guanidinobenzimidazole (2GBI)] (Hong et al., 2014). Unfortunately, they bind only from the intracellular side, therefore, they only work when the channel is open, making their application highly impractical (DeCoursey, 2015). We wonder whether the specific HVCN1 antibody characterized in this work can bind to the extracellular side of the plasma membrane of cells, and find that without cell membrane permeated by detergent, the HVCN1 antibody can bind to the extracellular side of the plasma membrane indeed. These data suggest that the HVCN1 antibody can be used to neutralize HVCN1. We show that myelin debris accumulation occurs not only within focal demyelination lesion but also in spinal ventral horn where spinal motor neuron degeneration takes place, suggesting that myelin debris accumulation is part of the pathology. Interestingly, we show when myelin was co-injected with the HVCN1 antibody, migration of microglia is significantly augmented *in vivo*. Importantly, neutralization of HVCN1 with antibody enhances myelin debris clearance in the brain *in vivo*. Together, these results provide a new approach to enhance myelin debris clearance. For translational purpose, how to make the HVCN1 antibody pass through the blood-brain barrier remains an obstacle to be overcome.

HVCN1 proton channel is unique among ion channels, and it conducts proton exclusively and only opens when acid extrusion will result (DeCoursey, 2015; Izquierdo et al., 2019). Expression of HVCN1 in microglia is conserved across species from mouse, marmoset, to human. In addition, OPC and oligodendrocyte in the mouse brain also express HVCN1 at a much lower level but astrocytes do not. The protein level of HVCN1 in the brain does not change with progressive aging, in both naturally aged and prematurely aged mice, and also in humans.

In summary, in this study, we identified a specific anti-HVCN1 antibody. We compared the relative abundance of HVCN1 at both mRNA and protein levels among various types of glial cells in the mouse brain. We found that in physiological conditions, microglia and BMDM express HVCN1 at the highest level among glial cells, while OPC and oligodendrocyte also express HVCN1 albeit at a much lower level, but astrocyte does not express HVCN1. High abundance of HVCN1 in microglia conserves across species from mouse, marmoset, to human. We further showed that upregulation of HVCN1 in microglia/macrophages is presented in multiple injuries and diseases of the CNS, reflecting the overactivation of HVCN1. Functionally, both genetic deletion of *HVCN1 in vitro* and neutralization of HVCN1 with antibody *in vivo* promotes cell migration. Our study couples the voltage-gated proton channel HVCN1 in microglia/macrophages to their migration, uncovering a new role for HVCN1 in the migration of microglia/macrophages. Importantly, neutralization of HVCN1 with antibody *in vivo* promotes myelin debris clearance capacity in microglia/macrophages, providing a potential new way to ameliorate the detrimental environment in the injured or diseased CNS by manipulating HVCN1 in microglia/macrophages.

## Data Availability Statement

The datasets presented in this study can be found in online repositories. The names of the repository and accession number can be found below: NCBI and the accession number is GSE183101 GSE183101 (https://www.ncbi.nlm.nih.gov/geo/query/acc.cgi?acc=GSE183101).

## Ethics Statement

The studies involving human participants were reviewed and approved by Medical Ethics Review Committee of Zhejiang University School of Medicine. Written informed consent to participate in this study was provided by the participants’ legal guardian/next of kin. The animal study was reviewed and approved by Experimental Animal Welfare Ethics Review Committee of Zhejiang University.

## Author Contributions

J-WZ perceived and supervised this project. J-WZ and FW designed the experiment. FW, X-RM, YW, D-XW, Y-CX, H-MG, and Z-JD performed the experiments. FW, X-RM, and YW analyzed the data. J-WZ, FW, X-RM, and YW wrote the manuscript. L-BW and H-LL provided some resources and discussion. All the authors have read and approved the final manuscript.

## Conflict of Interest

The authors declare that the research was conducted in the absence of any commercial or financial relationships that could be construed as a potential conflict of interest.

## Publisher’s Note

All claims expressed in this article are solely those of the authors and do not necessarily represent those of their affiliated organizations, or those of the publisher, the editors and the reviewers. Any product that may be evaluated in this article, or claim that may be made by its manufacturer, is not guaranteed or endorsed by the publisher.

## Footnotes

1
http://zjubrainbank.zju.edu.cn
