# Supplementary material for: Neutralization of Hv1/HVCN1 With Antibody Enhances Microglia/Macrophages Myelin Clearance by Promoting Their Migration in the Brain
Source: Front Cell Neurosci. 2021 Oct 22;15:768059. doi: 10.3389/fncel.2021.768059 (PMC8570284; doi:10.3389/fncel.2021.768059)
Supplement: Supplementary file 1 [file Data_Sheet_1.pdf]

Supplementary Material

1 Supplementary Figures and Tables

1.1 Supplementary Figures

Supplementary Figure 1

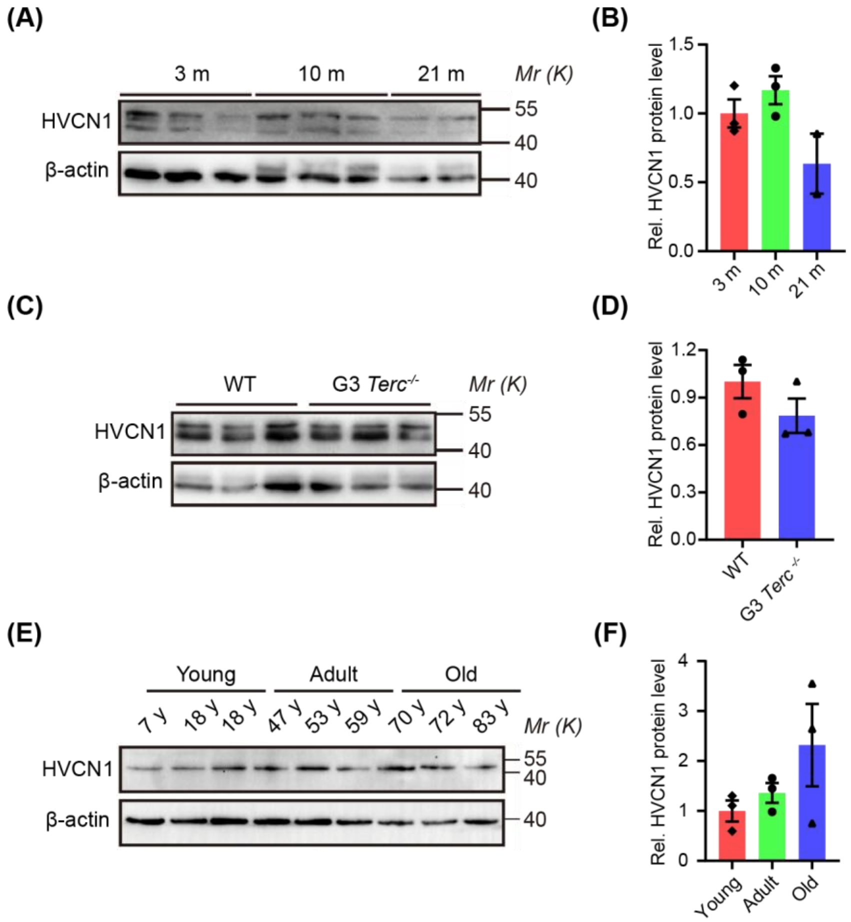

Supplementary Figure 1. The Protein Level of HVCN1 in the Brain Does Not Change with Aging. (A, B) Western blot images **(A)** and quantification of the density **(B)** of HVCN1 in the mouse brain does not change with aging (n = 3, 3, 2/group). **(C, D)** Western blot images **(C)** and quantification of the density **(D)** of HVCN1 in the brain show that HVCN1 does not change in G3 *Terc*$^{-/-}$ premature aged mouse brain (n = 3/group). **(E, F)** Immunoblot images **(E)** and quantification of the density **(F)** of HVCN1 in the human brain show that the protein level of HVCN1 does not change with aging in human brain (n = 3/age group). Data are presented as mean ± SEM. Statistical tests used: one-way ANOVA with Tukey's multiple comparison post-test **(B, F)** or unpaired two-tailed t-test **(D)**.

Supplementary Figure 2

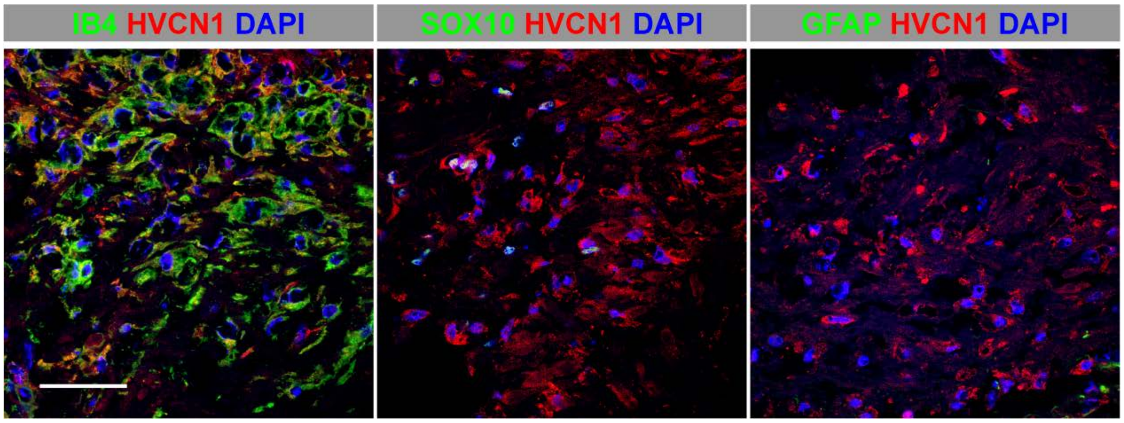

Supplementary Figure 2. HVCN1 is Mainly Expressed in Microglia/Macrophages in LPC-induced Demyelination Lesion in the Mouse Brain. In LPC-induced focal demyelination lesion in corpus callosum of the mouse brain, immunofluorescence images show that HVCN1 (red) is expressed mainly in IB4^{+} microglia/macrophages (green, left), scarcely in SOX10^{+} oligodendrocyte lineage cells (green, middle) but not in GFAP^{+} astrocytes (green, right). Scale bar = 50 μm.

Supplementary Figure 3

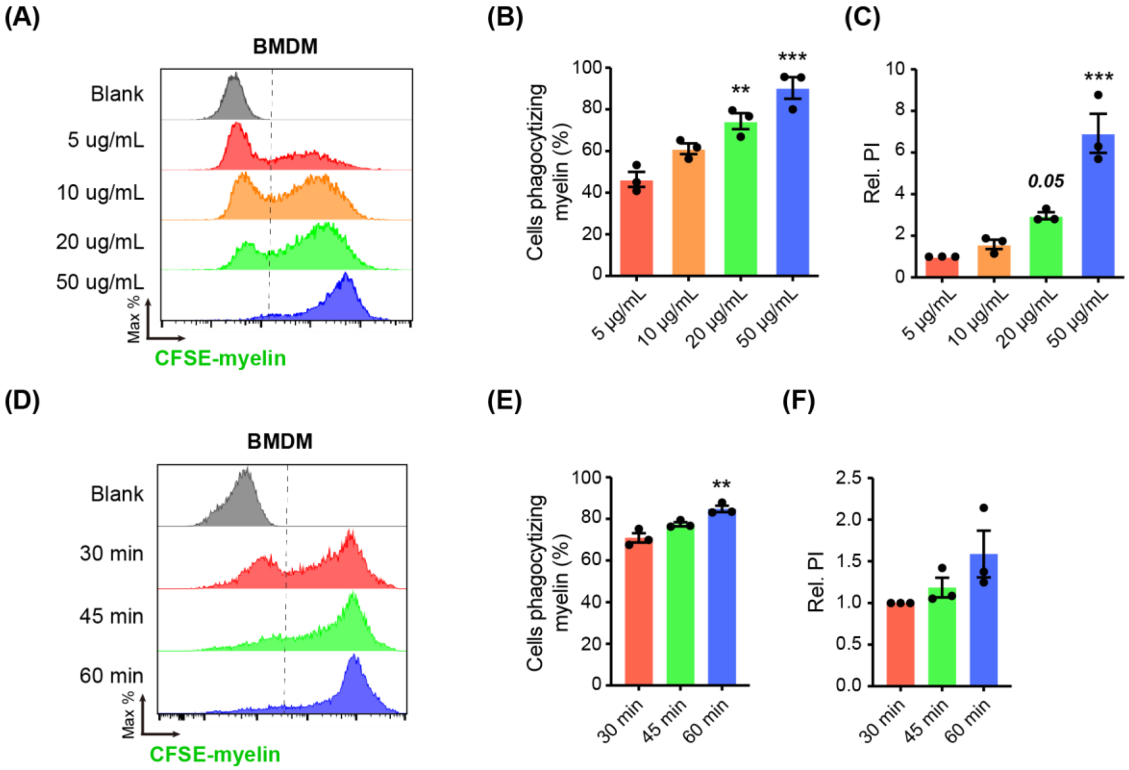

Supplementary Figure 3. Optimization of Myelin Phagocytosis Assay in BMDM Cells. (A-C) CFSE-labelled myelin phagocytosis assay in BMDM to test different doses of myelin. Flow cytometry **(A)** and quantification of the proportion of cells phagocytizing myelin **(B)** and the relative phagocytic

index **(C)** (n = 3). **(D-F)** CFSE-labelled myelin phagocytosis assay in BMDM to test different periods of time. Flow cytometry **(D)** and quantification of the proportion of phagocytotic cells **(E)** and the relative phagocytic index **(F)** (n = 3). Data are presented as mean ± SEM. Statistical tests: one-way ANOVA with Dunnett's multiple comparison post-test **(B, C, E, F)**; * $p < 0.05$, ** $p < 0.01$, *** $p < 0.001$.

Supplementary Figure 4

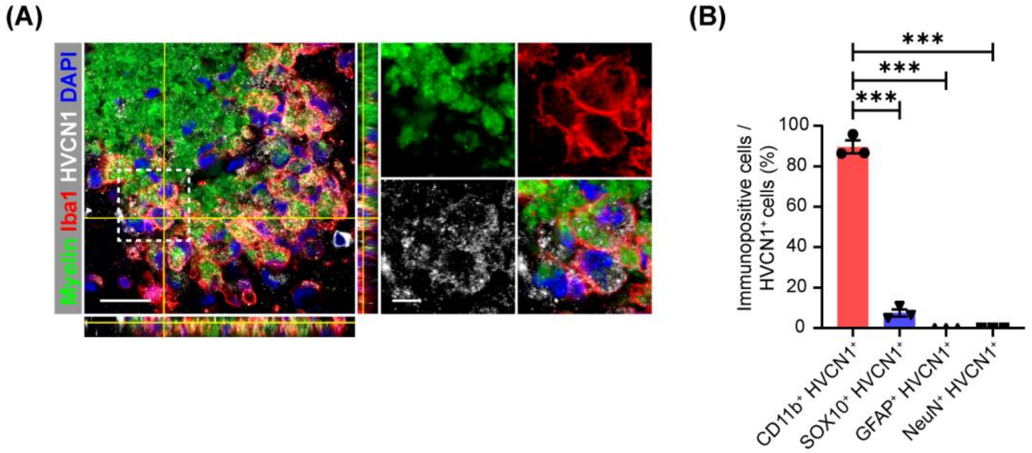

Supplementary Figure 4. Microglia account for the overwhelming majority of HVCN1$^+$ cells within the myelin injection site in the brain. (A) Representative images show that Iba1$^+$ (red) microglia express HVCN1 (gray) and phagocytize myelin (green). Scale bar = 20 µm in the left image while 5 µm in the right images in A. **(B)** Quantification graph shows that HVCN1$^+$ cells within the myelin injection area are predominantly microglia, scarcely oligodendrocytes, but not astrocyte or neuron (n = 3/group). Data are presented as mean ± SEM. Statistical tests: one-way ANOVA with Dunnett's multiple comparison post-test; *** p < 0.001.

Supplementary Figure 5

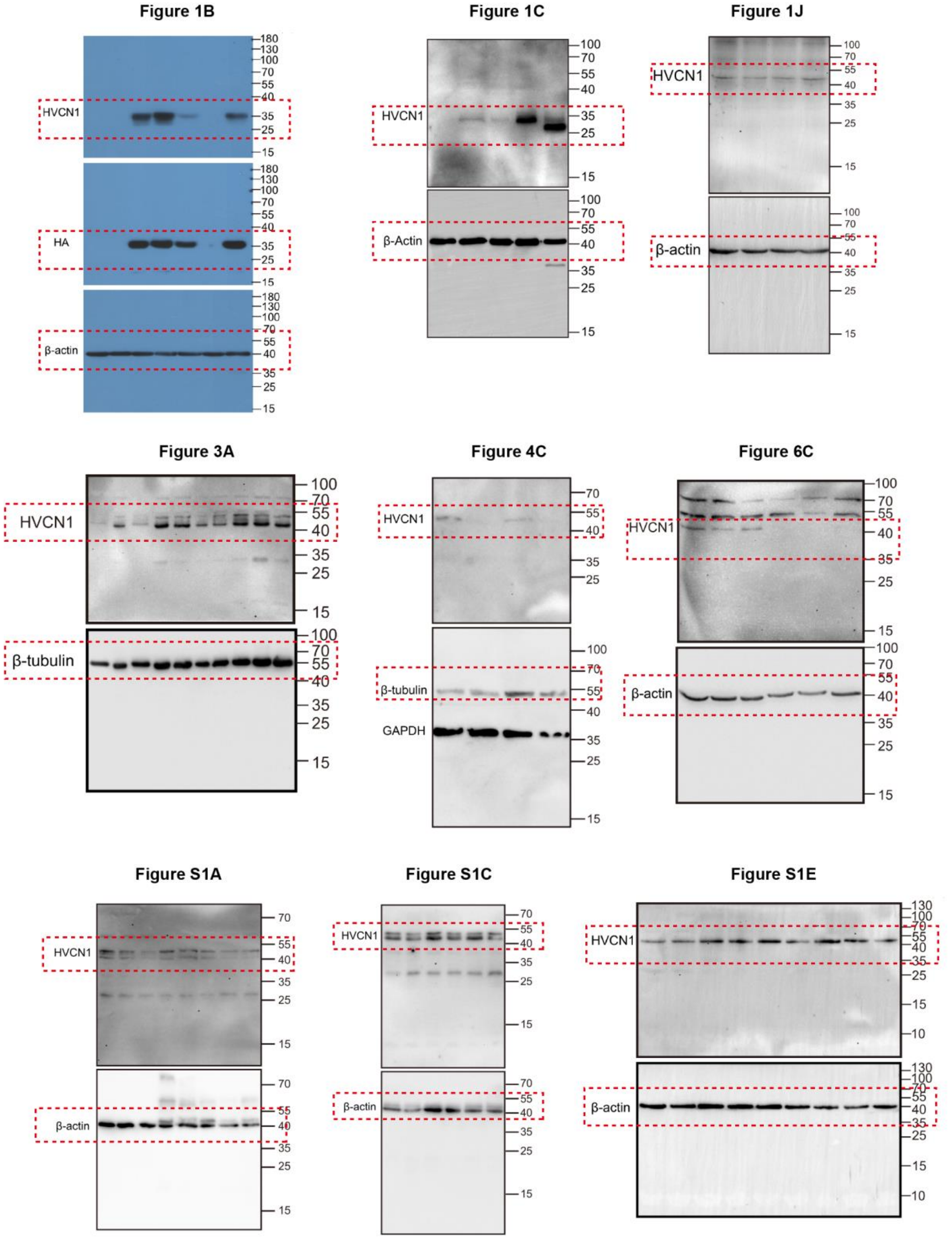

Supplementary Figure 5. Unprocessed Western blots.

1.2 Supplementary Tables

Supplementary Table 1. The Detailed Information of the Human Brain Samples.

Donor Number	Gender	Age (y)	Sample origin	Post mortem recovery delay	Diagnosis
2017CBB036	Female	7	cingulate gyrus	5h50m	Acute lymphocytic leukemia
2015CBB29	Male	18	cingulate gyrus	5h	Lower left pneumonia, progressive muscular dystrophy
2016CBB013	Male	18	cingulate gyrus	14h45m	Myasthenia gravis
2017CBB041	Female	47	cingulate gyrus	4h25m	Lung, liver, spleen, and abdominal cavity metastases after sigmoid colon cancer surgery
2016CBB0019	Male	53	cingulate gyrus	8h	Postoperative gastric cancer with recurrence and metastasis
2017CBB022	Male	59	cingulate gyrus	9h55m	Gastric cancer lymph node liver metastasis stage 4
2017CBB025	Female	70	cingulate gyrus	3h20m	Respiratory failure, malignant tumor of stomach
2015CBB25	Male	72	Prefrontal cortex	17h	No obvious disease
2017CBB011	Male	83	cingulate gyrus	24h40m	Gum cancer, prostate cancer, chronic obstructive pulmonary disease

Supplementary Table 2. Statistical methods, results and sample size related to each figure.

Figure	Statistical method	Factors	Statistical results	*Post hoc* method	*Post hoc* results	Sample size
1K	Unpaired two-tailed t test		Microglia vs. Cortex: t = 3.59, df = 13, p = 0.003			Cortex, n = 5; Microglia, n = 10
2B	Unpaired two-tailed t test		LPC lesion vs. Non-lesion: t = 9.695, df = 6, p < 0.0001			4 samples per group
2D	Unpaired two-tailed t test		Arg1+ vs. iNOS+: t = 17.75, df = 6, p < 0.0001			4 samples per group
2E	Unpaired two-tailed t test		Arg1+ vs. iNOS+: t = 1.534, df = 6, p = 0.1760			4 samples per group
2G	Unpaired two-tailed t test		LPS vs. PBS: t = 4.620, df = 4, p = 0.0099			3 samples per group
3B	Unpaired two-tailed t test		$SOD1^{G93A}$ vs. B6SJL: t = 2.098, df = 8, p = 0.0692			5 samples per group

Figure	Statistical method	Factors	Statistical results	*Post hoc* method	*Post hoc* results	Sample size
3D	Unpaired two-tailed t test		$SOD1^{G93A}$ vs. B6SJL: t = 4.722, df = 4, *p* = 0.0092			3 samples per group
3F	Unpaired two-tailed t test		$SOD1^{G93A}$ vs. B6SJL: t = 22.22, df = 4, *p* < 0.0001			3 samples per group
3H	Unpaired two-tailed t test		R6/2 vs. WT: t = 3.210, df = 4, *p* = 0.0326			3 samples per group
3I	Unpaired two-tailed t test		HD vs. Control: t = 3.340, df = 24, *p* = 0.0027			Control, n = 14; HD, n = 12
4E	Unpaired two-tailed t test		$HVCN1^{-/-}$ vs. WT: t = 4.365, df = 8, *p* = 0.0024			5 samples per group
5C	Paired two-tailed t test	Core area, DAPI / mm^2	Anti-HVCN1 vs. IgG: t = 1.697, df = 8, *p* = 0.0640			9 pairs
5C	Paired two-tailed t test	Core area, CD68 positive cells / mm^2	Anti-HVCN1 vs. IgG: t = 4.740, df = 8, *p* = 0.0007			9 pairs

Figure	Statistical method	Factors	Statistical results	*Post hoc* method	*Post hoc* results	Sample size
5C	Paired two-tailed t test	Core area, CD68 positive cells (%)	Anti-HVCN1 vs. IgG: t = 3.176, df = 8, p = 0.0065			9 pairs
5C	Paired two-tailed t test	Peripheral area, DAPI / mm^2	Anti-HVCN1 vs. IgG: t = 1.157, df = 8, p = 0.1403			9 pairs
5C	Paired two-tailed t test	Peripheral area, CD68 positive cells / mm^2	Anti-HVCN1 vs. IgG: t = 3.436, df = 8, p = 0.0044			9 pairs
5C	Paired two-tailed t test	Peripheral area, CD68 positive cells (%)	Anti-HVCN1 vs. IgG: t = 4.489, df = 8, p = 0.0010			9 pairs
6E	Two-way ANOVA	WT vs. HVCN1$^{-/-}$; Time	Interaction: F(3, 16) = 1.489, p = 0.2554. Time: F (3, 16) = 38.94, p < 0.0001. Gene: F (1, 16) = 0.2258, p = 0.6410.	Sidak's multiple comparisons test	1 h: WT vs. HVCN1$^{-/-}$, p = 0.5015; 2 h: WT vs. HVCN1$^{-/-}$, p = 0.9997; 3 h: WT vs. HVCN1$^{-/-}$, p = 0.8418; 4 h: WT vs. HVCN1$^{-/-}$, p = 0.6281.	3 samples per group

Figure	Statistical method	Factors	Statistical results	*Post hoc* method	*Post hoc* results	Sample size
6F	Two-way ANOVA	WT vs. HVCN1$^{-/-}$; Time	Interaction: F (3, 16) = 0.1811, *p* = 0.9077.<br><br>Time: F (3, 16) = 18.91, *p* < 0.0001.<br><br>Gene: F (1, 16) = 0.002697, *p* = 0.9592.	Sidak's multiple comparisons test	1 h: WT vs. HVCN1$^{-/-}$, *p* = 0.9801; 2 h: WT vs. HVCN1$^{-/-}$, *p* = 0.9968; 3 h: WT vs. HVCN1$^{-/-}$, *p* = 0.9999; 4 h: WT vs. HVCN1$^{-/-}$, *p* = 0.9887.	3 samples per group
6H	Unpaired two-tailed t test		HVCN1 vs. mCherry: t = 0.8453, df = 4, *p* = 0.4455			3 samples per group
6I	Unpaired two-tailed t test		HVCN1 vs. mCherry: t = 2.127, df = 4, *p* = 0.1006			3 samples per group
7C	Unpaired two-tailed t test		HVCN1$^{-/-}$ vs. WT: t = 4.308, df = 4, *p* = 0.0126			3 samples per group
7D	Unpaired two-tailed t test		HVCN1$^{-/-}$ vs. WT: t = 7.307, df = 4, *p* = 0.0019			3 samples per group

Figure	Statistical method	Factors	Statistical results	*Post hoc* method	*Post hoc* results	Sample size
7F	Unpaired two-tailed t test		HVCN1 vs. mCherry: t = 1.848, df = 4, p = 0.1383			3 samples per group
7G	Unpaired two-tailed t test		HVCN1 vs. mCherry: t = 0.4524, df = 4, p = 0.6744			3 samples per group
8C	Unpaired two-tailed t test		siHVCN1 vs. siNC: t = 1.013, df = 4, p = 0.3684			3 samples per group
8D	Unpaired two-tailed t test		siHVCN1 vs. siNC: t = 1.422, df = 4, p = 0.2282			3 samples per group
8F	Unpaired two-tailed t test		siHVCN1 vs. siNC: t = 0.02009, df = 8, p = 0.9845			5 samples per group
8G	Unpaired two-tailed t test		siHVCN1 vs. siNC: t = 1.120, df = 8, p = 0.2952			5 samples per group

Figure	Statistical method	Factors	Statistical results	*Post hoc* method	*Post hoc* results	Sample size
8I	Unpaired two-tailed t test		siHVCN1 vs. siNC: t = 0.4910, df = 12, p = 0.6323			7 samples per group
8J	Unpaired two-tailed t test		siHVCN1 vs. siNC: t = 1.59, df = 12, p = 0.1378			7 samples per group
9B	Unpaired two-tailed t test		LPC lesion vs. Non-lesion: t = 14.37, df = 4, p = 0.0001			3 samples per group
9D	Unpaired two-tailed t test		$SOD1^{G93A}$ vs. B6SJL: t = 12.51, df = 4, p = 0.0002			3 samples per group
10E	Paired two-tailed t test		Anti-HVCN1 vs. IgG: t = 2.665, df = 8, p = 0.0143			9 pairs
10F	Paired two-tailed t test		Anti-HVCN1 vs. IgG: t = 3.703, df = 8, p = 0.0030			9 pairs

Figure	Statistical method	Factors	Statistical results	*Post hoc* method	*Post hoc* results	Sample size
S1B	One-way ANOVA	Condition	F (2, 5) = 3.937, *p* = 0.0940.	Tukey's multiple comparisons test	3 m vs. 10 m, *p* = 0.6177; 3 m vs. 21 m, p=0.2298; 10 m vs. 21 m, *p* = 0.0832	3 m, n = 3; 10 m, n = 3; 21 m, n = 2.
S1D	Unpaired two-tailed t test		G3 $Terc^{-/-}$ vs. WT: t = 1.423, df = 4, *p* = 0.2278			3 samples per group
S1F	One-way ANOVA	Condition	F (2, 6) = 1.821, *p* = 0.2410.	Tukey's multiple comparisons test	Young vs. Adult, *p* = 0.8715; Young vs. Old, p=0.2339; Adult vs. Old, *p* = 0.4258	3 samples per group
S3B	One-way ANOVA	Condition	F (3, 8) = 22.91, *p* = 0.0003.	Dunnett's multiple comparisons test	5 μg/mL vs. 10 μg/mL, *p* = 0.0698; 5 μg/mL vs. 20 μg/mL, p=0.0025; 5 μg/mL vs. 50 μg/mL, *p* = 0.0001	3 samples per group
S3C	One-way ANOVA	Condition	F (3, 8) = 29.52, *p* = 0.0001.	Dunnett's multiple comparisons test	5 μg/mL vs. 10 μg/mL, *p* = 0.7363; 5 μg/mL vs. 20 μg/mL, p=0.0535; 5 μg/mL vs. 50 μg/mL, *p* < 0.0001	3 samples per group
S3E	One-way ANOVA	Condition	F (2, 6) = 17.27, *p* = 0.0032.	Dunnett's multiple comparisons test	30 m vs. 45 m, *p* = 0.0555; 30 m vs. 45 m, *p* = 0.0019.	3 samples per group

Figure	Statistical method	Factors	Statistical results	*Post hoc* method	*Post hoc* results	Sample size
S3F	One-way ANOVA	Condition	F (2, 6) = 2.953, p = 0.1280.	Dunnett's multiple comparisons test	30 m vs. 45 m, p = 0.6897; 30 m vs. 45 m, p = 0.0943.	3 samples per group
S4B	One-way ANOVA	Condition	F (3, 8) = 587.9, $p < 0.0001$.	Dunnett's multiple comparisons test	$CD11b^{+}HVCN1^{+}$ vs. $SOX10^{+}HVCN1^{+}$, $p < 0.0001$; $CD11b^{+}HVCN1^{+}$ vs. $GFAP^{+}HVCN1^{+}$, $p < 0.0001$; $CD11b^{+}HVCN1^{+}$ vs. $NeuN^{+}HVCN1^{+}$, $p < 0.0001$;	3 samples per group
